# Single-cell transcriptomics links malignant T cells to the tumor immune landscape in cutaneous T cell lymphoma

**DOI:** 10.1038/s41467-022-28799-3

**Published:** 2022-03-03

**Authors:** Xiangjun Liu, Shanzhao Jin, Simeng Hu, Ruoyan Li, Haihao Pan, Yi Liu, Pan Lai, Deshu Xu, Jingru Sun, Ziyang Liu, Yumei Gao, Yifan Zhao, Fengjie Liu, Yu Xiao, Yingyi Li, Yujie Wen, Zhuojing Chen, Bufang Xu, Yuchieh Lin, Menglong Ran, Qianxi Li, Shuxia Yang, Hang Li, Ping Tu, Muzlifah Haniffa, Sarah A. Teichmann, Fan Bai, Yang Wang

**Affiliations:** 1 Department of Dermatology and Venerology, Peking University First Hospital, Beijing, 100034 China; 2 Beijing Key Laboratory of Molecular Diagnosis on Dermatoses, Beijing, 100034 China; 3 National Clinical Research Center for Skin and Immune Diseases, Beijing, 100034 China; 4 Biomedical Pioneering Innovation Center (BIOPIC), and School of Life Sciences, Peking University, Beijing, 100871 China; 5 BioMap (Beijing) Intelligence Technology Limited, Block C Information Center Haidian District, Beijing, 100086 China; 6 Academy for Advanced Interdisciplinary Studies (AAIS), and Peking University–Tsinghua University–National Institute of Biological Sciences Joint Graduate Program (PTN), Peking University, Beijing, 100871 China; 7 Wellcome Sanger Institute, Wellcome Genome Campus, Hinxton, Cambridge, CB10 1SA UK; 8 Biosciences Institute, Newcastle University, Newcastle upon Tyne, NE2 4HH UK; 9 Department of Dermatology and NIHR Newcastle Biomedical Research Centre, Newcastle Hospitals NHS Foundation Trust, Newcastle upon Tyne, NE2 4LP UK; 10 Theory of Condensed Matter Group, Cavendish Laboratory/Department of Physics, University of Cambridge, Cambridge, CB3 0HE UK; 11 Beijing Advanced Innovation Center for Genomics (ICG), Peking University, Beijing, China; 12 Center for Translational Cancer Research, First Hospital, Peking University, Beijing, 100871 China

**Keywords:** Tumour immunology, Skin cancer, Tumour heterogeneity, Oncogenesis, T-cell lymphoma

## Abstract

Cutaneous T cell lymphoma (CTCL) represents a heterogeneous group of non-Hodgkin lymphoma distinguished by the presence of clonal malignant T cells. The heterogeneity of malignant T cells and the complex tumor microenvironment remain poorly characterized. With single-cell RNA analysis and bulk whole-exome sequencing on 19 skin lesions from 15 CTCL patients, we decipher the intra-tumor and inter-lesion diversity of CTCL patients and propose a multi-step tumor evolution model. We further establish a subtyping scheme based on the molecular features of malignant T cells and their pro-tumorigenic microenvironments: the T_CyEM_ group, demonstrating a cytotoxic effector memory T cell phenotype, shows more M2 macrophages infiltration, while the T_CM_ group, featured by a central memory T cell phenotype and adverse patient outcome, is infiltrated by highly exhausted CD8^+^ reactive T cells, B cells and Tregs with suppressive activities. Our results establish a solid basis for understanding the nature of CTCL and pave the way for future precision medicine for CTCL patients.

## Abstract (web summary)

Cutaneous T cell lymphomas (CTCL) are still poorly characterised at the molecular and single-cell level. Here, the authors analyse CTCL patient samples using single-cell RNA-seq, TCR and whole-exome sequencing, revealing the molecular profiles of malignant T cells and their association with the microenvironment and clinical outcomes.

## Introduction

Cutaneous T cell lymphomas (CTCL) is a heterogeneous group of extranodal non-Hodgkin’s lymphomas characterized by cutaneous infiltration of clonal malignant T cells. Mycosis fungoides (MF), Sézary syndrome and primary cutaneous anaplastic large cell lymphoma (pcALCL) constitute the majority of CTCLs and are believed to be derived from skin-homing mature T cells. MF begins with multiple patches, plaques and tumors in the skin, and it may involve lymph nodes, peripheral blood and viscera in the advanced stage, leading to a poor prognosis^1^. Sézary syndrome, the leukemic form of CTCL, is characterized by generalized skin erythema with leukemic malignant T cells in the blood, while pcALCL is characterized by single or multiple skin tumors and a tendency for systemic involvement in the late stage. These three CTCL variants are highly related and have overlapping clinical and immunophenotypic features^2,3^. These entities frequently coexist in the same patient, whether they develop simultaneously or as a secondary lymphoma^4^. Given the numerous shared and disparate properties of CTCL variants, it remains unclear whether these entities are distinct disorders or a continuum with a degree of genetic diversity and varied microenvironments.

Despite tremendous progression in the understanding of CTCL in recent decades, the origin and nature of malignant T cells remain to be defined. Unlike solid tumors, CTCL starts with multi-focal skin lesions, which leads to controversies regarding the tumor origin and evolution pattern of CTCL. Previous studies suggested that neoplastic T cells in CTCL originated from mature, monoclonal, skin-resident memory T cells^5^. However, recent evidence indicates that malignant T cells are derived from circulating immature precursor cells, which seed the skin and evolve into clonally heterogeneous lymphomas^6–8^. This discrepancy is largely due to the difficulty in discriminating true malignant T cells from benign reactive T cells in CTCL skin lesions, since there are no specific markers to define malignant T cells, which also results in delayed diagnosis and poor prognosis for CTCLs.

Owing to the poor understanding of disease pathogenesis, effective treatments for CTCL are limited, and the treatment response varies greatly among patients. CTCL generally exhibits an indolent course, but a portion of patients progresses rapidly and exhibit widespread disease beyond the skin despite aggressive treatment, highlighting the underlying heterogeneity of this disease. Moreover, CTCL patients frequently show inter-lesional diversity in treatment responses, adding to the complexity of the clinical challenge of the disease and often creating obstacles to achieving effective therapy^9^. However, the molecular mechanisms underlying this inter-lesional diversity remain to be defined.

The difficulty in distinguishing malignant T cells from reactive T cells presents an obstacle to understand the immune landscape of the tumor microenvironment (TME) in CTCL. Although studies have demonstrated that tumor infiltrating immune cells, including B cells and M2 macrophages, participate in the pathogenesis and persistence of CTCL through diverse cytokines and chemokines, the complex interplay between malignant T cells and TME compartments remains poorly understood^10,11^. In particular, malignant T cells preserve certain immunological features of mature T cells, and thus are active in cytokine production. Elucidating how malignant T cells redefine and alter the TME, as well as clarifying the landscapes and properties of the multicellular ecosystem in CTCL, will create new opportunities for the development and application of immunotherapies.

Here, we employed single-cell RNA sequencing (scRNA-seq) and paired single-cell T cell receptor (TCR) sequencing on CTCL skin tumors. Malignant T cells were defined by copy number variations (CNVs) and matched TCRα and TCRβ clonotypes at the single-cell level. With this approach, we determined the mono-clonal nature of CTCL and characterized the temporal and topological subclonal evolutionary process of malignant T cells, revealing a high degree of tumoral cellular heterogeneity. The genetic basis of this intratumor heterogeneity (ITH) was defined by paired whole exome sequencing (WES). We generated a molecular subtyping scheme based on the intrinsic identity of malignant T cells across CTCL variants, and we deciphered the complex crosstalk among immune cells within the TME for each subtype.

## Results

### Single-cell transcriptome analysis defines malignant T cells and reveals inter-tumor transcriptional heterogeneity in malignant T cells

**Fig. 1:**
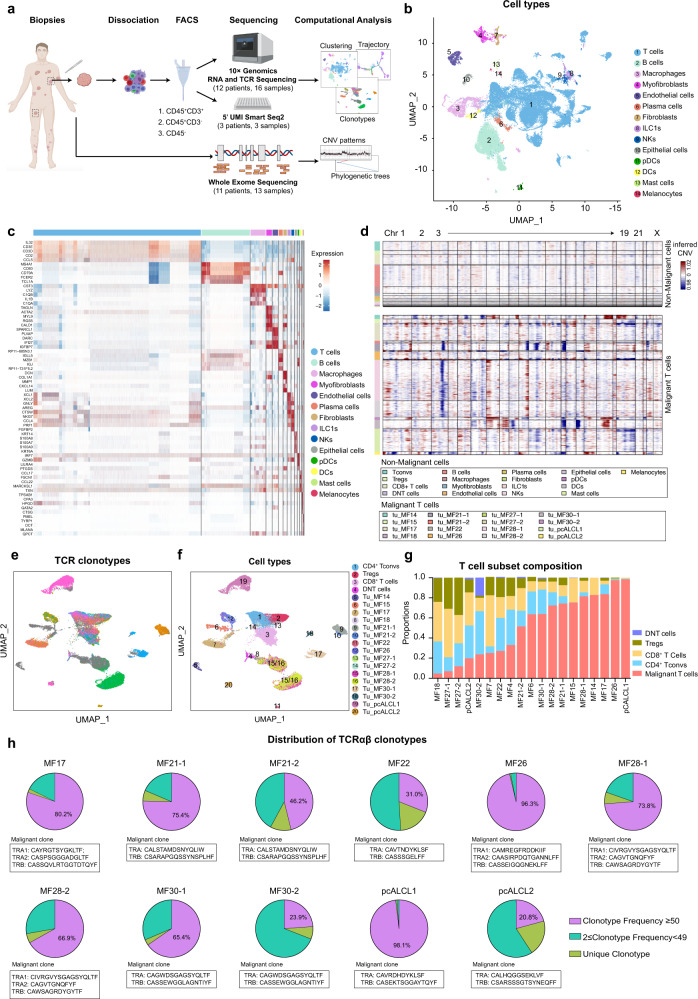
Single-cell transcriptional profiling of 19 cutaneous T cell lymphoma samples. **a** Workflow of tumor collection, single-cell dissociation, cell sorting, and computational analysis for scRNA-seq data and whole exome sequencing (WES) data. Among 16 samples subjected to the 10× Genomics method, the 10× 3′v3 method was applied to two samples from two patients, and the 10× 5′v2 method coupled with TCR V(D)J sequencing was applied to the remaining 14 samples from 10 patients. **b** UMAP plot shows 58, 926 high-quality cells from the 10× Genomics dataset. Fourteen cell types are defined by cell-specific markers. Each dot represents a single cell colored by cell type as annotated. ILC1s, type 1 innate lymphoid cells. NKs, natural killer cells. pDCs, plasmacytoid dendritic cells. DCs, dendritic cells. **c** Heatmap shows the expression of the top five signature genes in each cell type from the 10× Genomics dataset. Expression is indicated as the z-score normalized log_2_ level (count+1). **d** Large-scale CNVs of single cells from all samples. CNVs were inferred from the 10× Genomic dataset. **e** UMAP plots show all T cells from the 10× Genomics dataset after re-clustering, with cells with TCR information shown in color. Each color represents a distinct TCR clonotype. **f** UMAP plots show all T cells from the 10× Genomics dataset after re-clustering, with each cell colored by cell type. DNT cells: double negative T cells without expression of either *CD4* or *CD8B*. Tu: tumor cells. Malignant T cell clusters are named by the prefix “Tu-” coupled with the sample ID. **g** The proportions of malignant T cells and reactive T cells in each sample. We selected 13 patients (except patients MF18 and MF27) corresponding to 16 samples with >80 malignant T cells for further malignant T cell analysis. **h** Pie charts show the distribution of TCRαβ clonotypes of all T cells in each sample based on clonal frequency. The paired CDR3α and CDR3β sequences with clonal frequency >50 (the dominant clonotype) in each sample are listed below the pie charts. Source data for (**c**) and (**g**) are provided in the Source Data file.

To systematically examine the cellular profiles of CTCL, we performed scRNA-seq on cells from 19 freshly dissociated CTCL skin samples (*n* = 15 patients, including 11 CD4^+^ MF, 2 CD8^+^ MF, and 2 pcALCLs) (Fig. 1a and Supplementary Data File 1). For 4 CD4^+^ MF patients (patients MF21, MF27, MF28 and MF30), two biopsies at different anatomic sites were obtained. All diagnoses were verified by two board-certified dermatopathologists and all biopsies were taken from thick plaques or tumors. Fluorescence-activated cell sorting (FACS) was utilized to enrich for several fractions; (i) CD45^+^CD3^+^ T cells, (ii) CD45^+^CD3^−^ immune cells and (iii) CD45^−^ non-immune cells (Fig. 1a and Supplementary Fig. 1a). To balance the gene coverage and cell productivity, we analyzed 3 samples (patients MF4, MF6 and MF7) using 5′ unique molecular identifier (UMI)-based Smart-Seq2 method, and 16 samples were subjected to a droplet-based 10× Genomics platform. For the 10× Genomics method, the single-cell 5′ reagent kit coupled with TCR V(D)J analyses was used to profile TCR clonotypes at single-cell resolution for 14 samples, while a single-cell 3′ reagent kit was used for 2 samples (Supplementary Data File 2). Whole exome sequencing was also performed on 13 CTCL skin samples from 11 patients.

Following stringent quality control (see Methods), a total of 60,324 cells were retained for subsequent analysis (Supplementary Data File 2). We performed unsupervised clustering and projected cells in two dimensions using uniform manifold approximation and projection (UMAP). We identified 14 major cell types based on the expression of canonical marker genes, including T cells, B cells, macrophages, plasma cells, natural killer (NK) cells, type 1 innate lymphoid cells (ILC1s), dendritic cells (DCs), plasmacytoid dendritic cells (pDCs), mast cells and skin-resident non-immune cells (including epithelial cells, endothelial cells, fibroblasts, myofibroblasts and melanocytes), from a total of 58, 926 cells in the 10× Genomics dataset (Fig. 1b, c)^12^, demonstrating a multicellular ecosystem in CTCL skin lesions. A similar pipeline was applied to the 5′ UMI Smart-Seq2 dataset (Supplementary Fig. 1b, c). We were able to capture more cells per skin sample using 10× Genomics (median of 2,143 cells versus 453 cells using Smart-Seq2) with both methods providing comparable median number of genes per cell (1,483 genes using 10× Genomics versus 1,578 genes using Smart-seq2) (Supplementary Fig. 1e and Supplementary Data File 2).

To differentiate malignant T cells from reactive T cells, TCR repertoires and large-scale chromosomal CNVs inferred from transcriptome sequencing were examined^9,12,13^. Malignant T cells were defined as presenting TCR clonal expansion coupled with apparent CNVs (Fig. 1d, e and Supplementary Fig. 1d, f). The CNV patterns of malignant T cell subsets were highly consistent with those generated from paired bulk whole exome sequencing (Supplementary Fig. 1g). As visualized in the UMAP plot of all T cells, reactive T cells, including conventional CD4^+^ T cells (Tconvs), CD4^+^ regulatory T cells (Tregs) and CD8^+^ T cells, were clustered by cell type, and the transcriptome profiles from each sample showed a high degree of overlap, whereas malignant T cells grouped into clear patient-specific clusters, demonstrating the remarkable inter-tumor heterogeneity of malignant T cells (Fig. 1f and Supplementary Fig. 1d and 2a).

The proportion of malignant T cells within the total T cell population was highly variable across samples, even between samples from the same patient, adding to the complexity of inter-tumor heterogeneity among CTCL lesions (Fig. 1g). After excluding MF18 and MF27, which had a limited number of malignant T cells (<80), we selected the remaining 16 samples from 13 patients for further malignant T cell analysis. In addition, among the benign reactive T cells without apparent CNVs and presenting a polyclonal phenotype, cells without expression of either *CD4* or *CD8B* were excluded from subsequent analysis (Fig. 1f).

By interrogating the paired TCRα and TCRβ repertories all T cells (*n* = 35,754), we showed that 79.7% (28,511/35,754) contained at least one productive TCRα or β chain and 73.6% (26318/35754) had both α and β chains (Supplementary Fig. 1h). We observed that malignant T cells from each sample harbored a unique clonotype (clonotype frequency > 210 in all samples), and biopsies from different anatomical compartments of the same patient shared the same TCR clonotype (Fig. 1h). In patients MF17, MF26 and MF28, there were two *TCRα* chains coupled with a single *TCRβ* chain in the malignant clone. Approximately 30% of mature αβ T cells express dual *TCRA* chains due to the absence of transcriptional allelic exclusion^14^. These results strongly supported the hypothesis of a monoclonal origin for CTCL^5^. Interestingly, in patient pcALCL1, in which CD4^+^ and CD8^+^ T cells were both predominant according to histological analysis, CD8^+^ T cells were defined as malignant T cells with clonal expansion and aberrant CNVs. Thus, pcALCL1 was reclassified as a CD8^+^ pcALCL, which has rarely been reported^9^.

Notably, we also observed a fraction of reactive T cells (no apparent CNVs) exhibited low-level clonal expansions in all samples (clonotype frequency 2–49), indicating an active T cell-mediated immune reaction in CTCL lesions (Fig. 1h).

To summarize, scRNA-seq analysis with TCR profiling unambiguously identified malignant T cells in CTCL samples and revealed a remarkable inter-tumor heterogeneity of malignant T cells.

### The constitutive activation/proliferation programs of malignant T cells in each patient determined the inter- and intra-tumor heterogeneity of CTCL

Concordant with the characteristics of neoplastic cells previously described in advanced CTCL, a lack of *CD7* expression was observed in malignant T cell subsets^15^ (Supplementary Fig. 2a). Malignant T cells showed variable expression of *SELPLG* (encoding CLA), which was indicative of a skin-homing T cell phenotype^5^. All CD4^+^ T cells, including both reactive and malignant T cells from all samples, presented a T-helper (Th)-2 skewing phenotype (defined by *GATA3* expression), consistent with previous findings that Th2 cytokines produced by malignant T cells repressed the Th1 immune response, fostered a Th2-biased phenotype in normal T helper cells, and impaired antitumor immunity in advanced CTCL^16^. In MF17 and MF22, malignant T cells displayed a Th1 (with *TBX21* expression) and Th2 phenotypes simultaneously. A concurrent Th17 phenotype (defined by *RORC* expression) was identified in pcALCL2, which was consistent with a previous report demonstrating a dual Th2-Th17 phenotype in a portion of pcALCLs^17^.

**Fig. 2:**
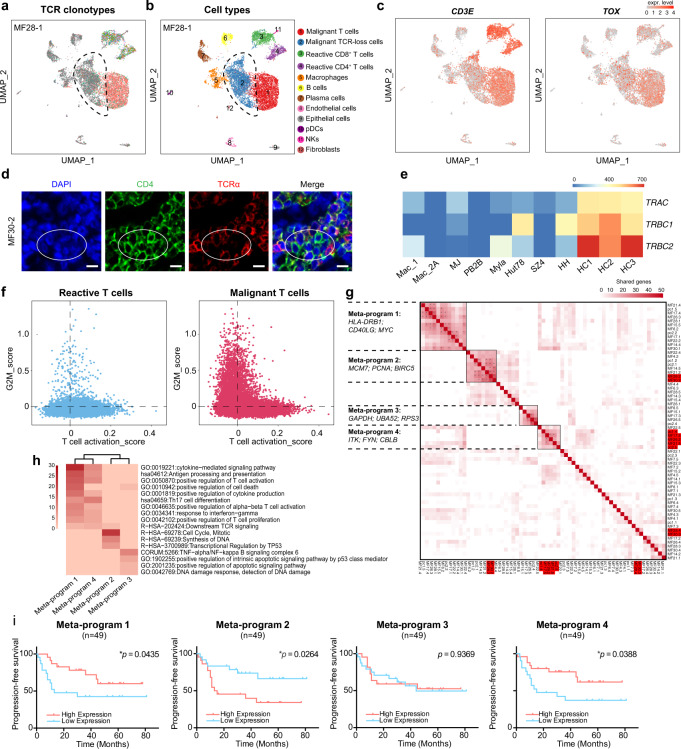
Malignant T cells displayed highly diversified transcriptional profiles. **a** UMAP plot shows all cells from one representative sample (MF28-1), with cells with TCR information shown in color. Each color represents a distinct TCR clonotype. The dashed circle denotes a malignant T cell subset featured by a loss of expression of the *TCRα* and *TCRβ* chains. **b** UMAP plot shows all cells from one representative sample (MF28-1), with cells colored by cell types, related to Fig. 2a. **c** UMAP plot shows the expression of *CD3E* and *TOX* in all cells from sample MF28-1. The color scale represents normalized expression. Gray to red: low to high expression. **d** Immunofluorescence staining demonstrates that CD4^+^ tumorous cells (green) exhibit low expression of TCRα (red), exemplified by sample MF30-2. DAPI (blue) was used to visualize cell nuclei. Scale bar = 10 μm. Results are representative of three different samples. **e** RNA sequencing data shows that the expression levels of *TRAC*, *TRBC1* and *TRBC2* are significantly deceased in CTCL lines in comparison with those of normal peripheral CD4^+^ T cells from three healthy controls. HC healthy control. **f** Scatterplots show the T cell activation and proliferation (G2M score) states of reactive T cells (left) and malignant T cells (right) from the 10× Genomics dataset. Blue dots represent reactive T cells and red dots represent malignant T cells. **g** Heatmap shows hierarchical clustering based on the number of genes shared by two programs (rows and columns) derived from NMF analysis in the 10× Genomics dataset. Each dot presents one program from individual patients. Four highly correlated meta-programs were identified based on a minimum of 10 shared genes between two programs. TCR loss clusters in each sample were highlighted in red. *, two programs share more than 20 genes. **h** Heatmap shows pathway enrichment of four meta-programs. **i** Progression-free survival (PFS) analysis of an independent cohort of 49 tumor-stage MF patients. Patients were stratified into low and high expression groups according to median values of scores corresponding to the gene signatures of four meta-programs. *P* values were calculated using the log-rank test. Source data for (**e**) and (**g**) are provided in the Source Data file.

In the malignant T cell subsets, a cell cluster characterized by a loss of TCR expression was identified in five of the patients (patients MF17, MF26, MF28, MF30 and pcALCL1) (Fig. 1e and Fig. 2a, b). In this cluster, only less than 20% of cells harbored productive *TCRA* and *TCRB* pairs, which showed the same clonotype with the TCR-competent malignant T cell cluster. This TCR-loss cluster existed exclusively in malignant T cell populations, but not in reactive T cells, and it was grouped individually in each sample instead of mixed into malignant T cell subsets as a “dropout” event, pointing to a distinct transcriptional identity of these cells. These TCR-loss clusters showed lower *CD3E* expression and *TOX* expression compared with other malignant T cells, as well as slightly different CNV patterns (Fig. 2c and Supplementary Fig. 2b). To confirm the existence of these clusters of cells, we performed immunofluorescence in corresponding MF skin lesions, which revealed scattered TCRα^low^ CD4^+^ T cells in clinical samples (Fig. 2d).

Down-regulation of TCR expression has been reported in aggressive T-cell lymphomas, including primary epitheliotropic intestinal T-cell lymphoma and primary cutaneous gamma-delta T cell lymphoma (PCGDTL)^18,19^. Some recent studies suggested the existence of a special TCR-silent type of lymphoma, while others proposed that this phenotype represented a common phenomenon of TCR instability^18,19^. T cell activation could trigger TCR complex downregulation in normal mature human T cells. Although such downregulation is mainly due to post-translational degradation of the TCR complex, down-regulation of *TCRα* and *TCRβ* mRNA expression does during this process^20,21^. To further confirm our finding, we measured TCR gene expression in a variety of CTCL cell lines using RNA sequencing. In comparison with normal peripheral CD4^+^ T cells from healthy volunteers, all CTCL cell lines showed remarkably decreased expression of *TRAC, TRBC1* and *TRBC2* (Fig. 2e), coinciding with our findings of transcriptional down-regulation of TCRs in a portion of CTCLs.

Malignant T cells in CTCL are unusual tumor cells because they possess the features of immune cells as well as neoplastic cells. To clarify the activation/proliferation states of malignant T cells, all T cells were plotted by previously defined gene signatures (see Methods). As expected, reactive T cells showed a range of T cell activation statuses with a low G2M score, while a subset of malignant T cells showed a high G2M score with an attenuated activation status, and another subset retained the activation status and showed a low G2M score (Fig. 2f). The discrete activation/proliferation states observed among malignant T cells indicated a high level of heterogeneity in CTCL.

To further explore intratumor transcriptional diversity, a non-negative matrix factorization (NMF) analysis was applied to malignant T cells from each sample (see Methods). On average, five programs from each sample were extracted to generate a total of 65 gene signatures, revealing high intratumor heterogeneity (ITH) in each sample (Supplementary Fig. 2c). Hierarchical clustering of these signatures revealed four main meta-programs, which indicated the collective behaviors of malignant T cells across the heterogeneous transcriptional spectrum of all tumors (Fig. 2g). The four meta-programs represented distinct functional signatures annotated by the top-ranking genes, including T cell signaling and activation (meta-program 1: *HLA-DRB1, CD69* and *MYC*; and meta-program 4: *ITK, FYN* and *CBLB*), cell cycle (meta-program 2: *MCM7, PCNA* and *BIRC5*) and cell metabolism (meta-program 3: *GAPDH, BUA52* and *RPS3*) (Fig. 2h). Each meta-program existed in multiple samples and represented a biological process operating in a subset of malignant T cells in each sample. Interestingly, meta-program 4 consisted of the TCR-loss clusters in each sample, except for MF28 and MF30. This phenomenon may indicate that the activation-induced TCR complex down-modulation frequently occurs in malignant T cells, and the molecular features of TCR-loss clusters demonstrate heterogeneity across patients. We also noticed that meta-program 3 was enriched with genes encoding ribosomal proteins. It may reflect the scRNA-seq preferentiality of detecting highly expressed genes, or suggesting a dysregulated ribosomal protein expression in malignant T cells^22^.

Next, we determined whether these four meta-programs were related to patient prognosis by evaluating the top-ranking genes in each meta-program in an independent cohort from our group of 49 tumor-stage MF patients with bulk RNA-seq data on skin tumors^23^. Notably, a high T cell activation signature (meta-program 1 and meta-program 4) was associated with a favorable prognosis, while a high proliferation signature (meta-program 2) predicted a poor patient outcome (Fig. 2i). The adverse prognostic value of meta-program 2 was further validated in an independent pcALCL cohort with 15 patients, while meta-program 1 and 4 showed no significant effect on clinical outcomes in this pcALCL cohort, which may be associated with the limited sample sizes (Supplementary Fig. 2d and Supplementary Data File 3). These results indicate that malignant T cells in CTCL showed high intra-tumor heterogeneity with regard to transcription and function, and the composition of activation/proliferation programs in each sample determined the clinical behavior of the particular case and patient prognosis.

### Inter-lesion divergence in CTCL is the result of a multi-step seeding process by monoclonal malignant T cells with parallel subclonal evolution

ITH provides a diverse genetic and epigenetic background for selection and cancer evolution. However, CTCL presents as multiple skin lesions at the very early stage of the disease. The manner in which the high transcriptional ITH of CTCL is related to the evolution and spreading of skin lesions remains undetermined. To explore the temporal and spatial spreading patterns of CTCL, we analyzed paired tumors obtained from different anatomical sites from 3 CD4^+^ MF patients (patients MF21, MF28 and MF30). Interestingly, the transcriptome heterogeneity increased along with the time interval of lesion development and the anatomical distance between lesions in individual patients.

**Fig. 3:**
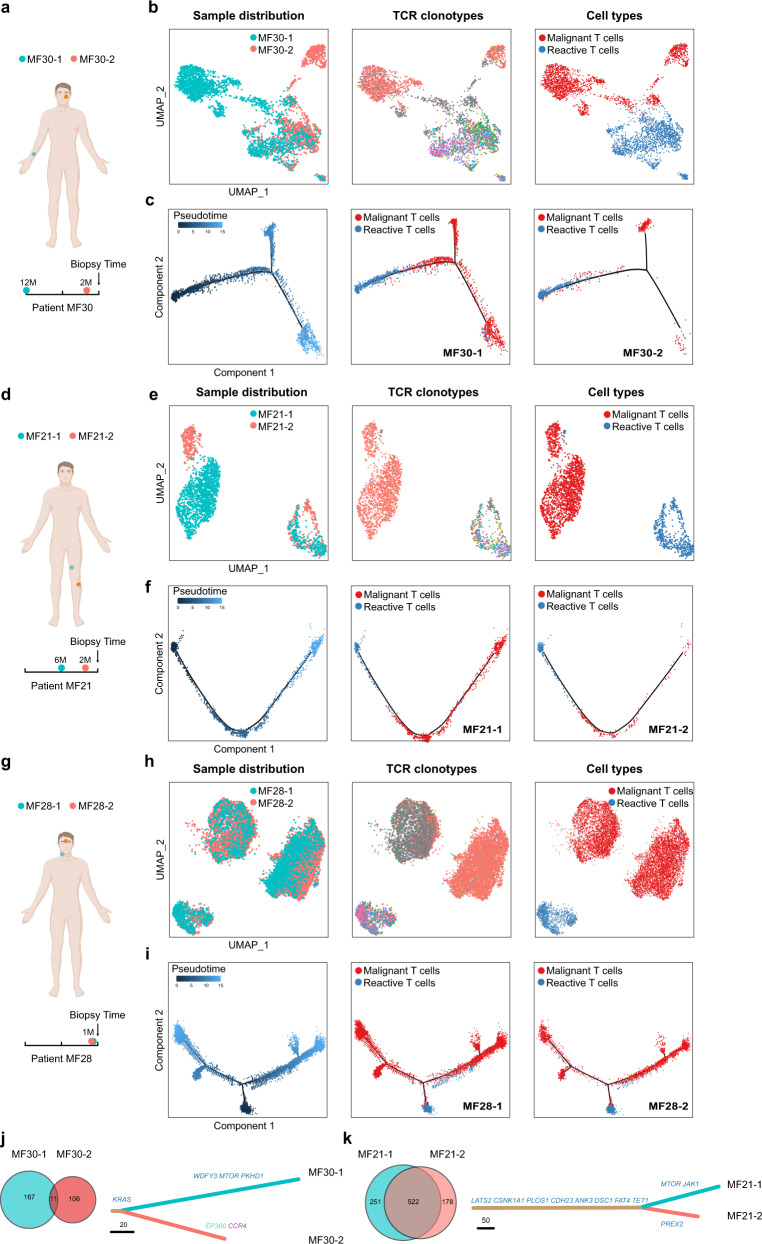
Inter-lesion diversity analysis of paired tumors from the same patient. **a**, **d**, **g** Schematic diagrams illustrate the anatomical sites and time intervals of paired tumors obtained from patients MF30 (**a**), MF21 (**d**) and MF28 (**g**). **b**, **e**, **h** UMAP plots showing all T cells from paired tumors from patients MF30 (**b**), MF21 (**e**) and MF28 (**h**), with cells colored by sample ID, TCR clonotypes (TCR information is shown in color and each color represents a distinct TCR clonotype) and cell types in sequence. **c**, **f**, **i** Pseudotime trajectory analysis of all T cells from paired tumors of patients MF30 (**c**), MF21 (**f**) and MF28 (**i**) inferred by Monocle 2, in which reactive T cells were selected as the start cells. Cell trajectories are further shown separately according to sample ID, with cells colored by cell types. **j**, **k** Venn diagrams show the numbers of nonsynonymous mutations of paired tumors from patient MF30 (**j**) and MF21 (**k**) inferred from WES data (left). Putative driver mutations are annotated on the phylogenetic trees of paired tumors (right). Missense mutations are in blue. Nonsense mutations are in green. Frameshift mutations are in purple. The length of each line is proportional to the number of nonsynonymous mutations. Source data for (**j**) and (**k**) are provided in the Source Data file.

In patient MF30 with a ten-year history of MF, in which the two tumors had the longest time interval and distance (MF30-1 developed on the right forearm 12 months prior to biopsy, whereas MF30-2 developed on the left cheek two months prior to biopsy), malignant T cells from two biopsies showed disparate transcriptome profiles, although they shared the same TCR clonotype, while the transcriptomes of benign T cells overlapped, as visualized by UMAP plot (Fig. 3a, b). Consistently, trajectory inference revealed that the evolutionary pathways of the malignant T cells from two separate lesions overlapped minimally along pseudotime (see Methods and Fig. 3c). To reveal the genetic background of this inter-lesion diversity, the single nucleotide variant (SNV) patterns of two samples were analyzed. As expected, the two tumors shared less than 10% non-synonymous mutations, and >90% mutations were private (Fig. 3j). The substantial degree of genetic divergence suggested that these two tumors arose from an early clone that was seeded to the skin of the entire body in the early stage of the disease, possibly before clinically detected lesions appeared, and subclonal tumor evolution subsequently occurred in parallel in the skin.

In contrast, in patient MF21 with a fifteen-year history of MF, whose samples were obtained from two tumors within the same anatomical compartment (MF21-1 developed above the left knee 6 months prior to biopsy, whereas MF21-2 developed below the left knee two months prior to biopsy), the transcriptome profiles of malignant T cells from the two lesions were closely related (Fig. 3d, e). Trajectory analysis revealed similar evolutionary dynamics, indicating the resemblance of transcriptional patterns between adjacent tumors (Fig. 3f). The phylogenetic relationship showed that >60% mutations were on the stem of the tree, illustrating a high degree of shared mutations (Fig. 3k). These results suggested a model in which the subclonal malignant T cells in a well-developed tumor may re-circulate and seed into adjacent skin areas and generate late-arising lesions.

This model was further confirmed by patient MF28 with a three-year history of MF, in which two newly developed tumors (less than 1 month old) at adjacent sites (MF28-1 was on the neck, whereas MF28-2 was between the eyebrows) exhibited highly overlapping transcriptional patterns and almost identical evolutionary trends (Fig. 3g–i). These two tumors might have arisen from the same subclone of an adjacent tumor. Consistently, this patient had folliculotropic MF and showed multiple skin tumors in the head and neck area. Interestingly, a similar pattern of ITH was observed in the two lesions, including the existence of a TCR-loss cluster in each sample (Fig. 3h). This phenomenon suggested that the late-arising lesions were seeded by a cluster of cells, which transmitted the genetic diversity for early-arising lesions via a mechanism analogous to consecutive seeding during metastasis of solid tumors^24^.

Our results suggested that tumor evolution was a multi-step seeding process. This dynamic evolution leads to the accumulation of ITH within each skin lesion, as well as inter-lesion diversity, and contributes to the heterogeneous clinical features and treatment response observed among different skin lesions in the same patient.

### The intrinsic features of malignant T cells suggest distinct CTCL tumor origins and determine diverse clinical outcomes

The complexity of CTCL in clinical settings lies in its various subtypes and classifications. The current classification scheme depends largely on clinical characteristics (e.g., folliculotropic, depigmented, or poikilodermic), cell morphology (e.g., large anaplastic cells or small-medium cells) and T cell types (e.g., CD4, CD8, and CD30), rather than on the intrinsic molecular features of malignant T cells. Since we have defined malignant T cells in each sample, we aimed to subtype the CTCL samples according to the transcriptome patterns of malignant T cells.

**Fig. 4:**
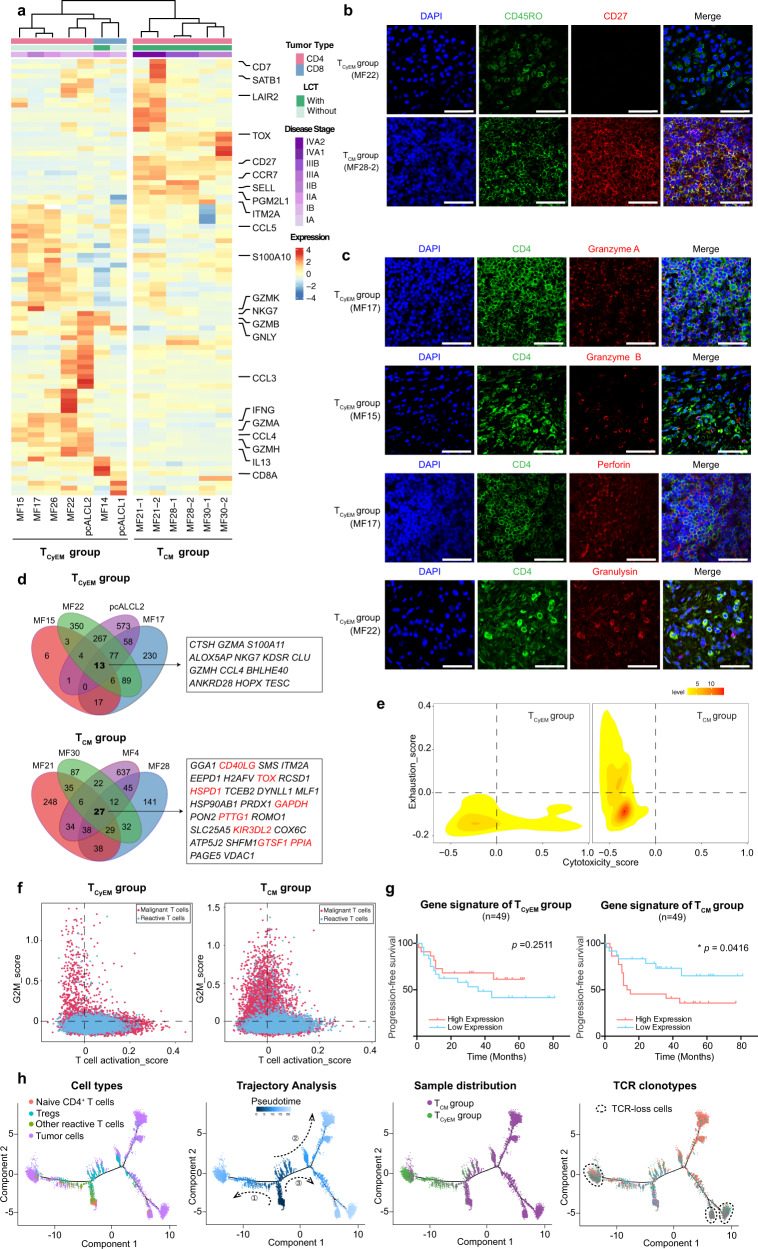
A molecular subtyping scheme for CTCL based on the transcriptomes of malignant T cells. **a** Heatmap of unsupervised hierarchical clustering showing the average expression of the DEGs between malignant T cells and their respective reactive CD4^+^ or CD8^+^ T cells in each sample from 13 patients in the 10× Genomics dataset (log_2_ fold change >1.5, *p* < 0.05). The bars above the heatmap show the tumor type, LCT information and disease stage of each patient. LCT, large cell transformation. **b** Immunofluorescence staining of CD45RO (green) and CD27 (red) on tumor samples from the T_CyEM_ and T_CM_ groups. DAPI (blue) was used to visualize cell nuclei. Scale bar = 50 μm. Results are representative of three different samples. **c** Immunofluorescence staining demonstrates that CD4^+^ tumorous cells (red) express several cytotoxic markers (red). DAPI (blue) was used to visualize cell nuclei. Scale bar = 50 μm. Results are representative of three different samples. **d** Venn diagrams illustrate the number of overlapping DEGs (log_2_ fold change >0.25, *p* < 0.05) of representative T_CyEM_ and T_CM_ patients. The gene signatures are listed. Genes previously reported to be upregulated in CTCL are highlighted in red. **e** 2D density plots show the cytotoxicity and exhaustion score of malignant T cells in the two groups from the 10× Genomics dataset. **f** Scatterplots show the T cell activation and proliferation states of all T cells in the two groups from the 10× Genomics dataset. Blue dots represent reactive T cells. Red dots represent malignant T cells. **g** PFS analysis of the 49 tumor-stage MF patients. Patients were stratified into low and high expression groups according to median values of scores corresponding to the gene signatures of the T_CyEM_ (left) and T_CM_ (right) groups identified in Fig. 4d. *P* values were calculated using the log-rank test. **h** Pseudotime trajectory analysis of all CD4^+^ T cells in CD4^+^ CTCL patients from the 10× Genomics dataset inferred by Monocle 2, with cells colored by cell types, pseudo-time, molecular subtypes and TCR clonotypes in sequence. Naive T cells were selected as the start cells. Source data for (**d**) are provided in the Source Data file.

We profiled the transcriptional expression patterns of all 25,919 malignant T cells and identified the differentially expressed genes (DEGs) between malignant T cells and their respective reactive CD4^+^ or CD8^+^ T cells in each sample from 13 patients. Unsupervised clustering analysis of these DEGs separated all samples from the 10× Genomic dataset into two groups independent of disease subtype (Fig. 4a). In one group, which contained CD4^+^ MF, CD8^+^ MF, CD4^+^ pcALCL, and CD8^+^ pcALCL, the malignant T cells exhibited an effector memory T phenotype, defined as CD45RO^+^, CD27^−^, CD62L (encoded by *SELL*)^-^ and CCR7^−^, except for one CD8^+^ MF (MF14), which exhibited a CD45RA^+^CD45RO^−^CD27^−^CD62L^−^CCR7^−^ phenotype, consistent with the effector memory T cells re-expressing CD45RA (T_EMRA_) as previously described (Fig. 4a, b and Supplementary Fig. 3a-b)^25,26^. Interestingly, malignant T cells in this group expressed a core set of cytotoxic markers, including *GZMA, GZMB, PRF1, GNLY* and *IFNG*, especially in all CD4^+^ MFs (Fig. 4a, c and Supplementary Fig. 3a).

In contrast, the other group, consisting of CD4^+^ MF samples, uniformly expressed high levels of CD45RO, CD62L, CD27 and CCR7, canonical markers of central memory T cells, whereas cytotoxic makers were absent (Fig. 4a, b and Supplementary Fig. 3a). A similar grouping pattern was seen in the 3 samples in the 5′ UMI Smart-Seq2 dataset (Supplementary Fig. 3c). Accordingly, we divided the 13 patients into a cytotoxic effector memory T cell (T_CyEM_) group and a central memory T cell (T_CM_) group. The T_CyEM_ group included four CD4^+^ MF patients, one CD4^+^ pcALCL patient, two CD8^+^ MF patients and one CD8^+^ pcALCL patient, while all five T_CM_ cases were CD4^+^ MF patients. Samples from different anatomical sites from one patient were included in the sample group, as expected.

To define the gene expression signature of each group, DEGs common to all samples in each group were identified. To maximize the representativeness of these gene signatures, we excluded samples with a limited number of DEGs. Thereby, 13-gene and 27-gene signatures were identified in the T_CyEM_ and T_CM_ groups, respectively (Fig. 4d). Malignant T cells in the T_CyEM_ group showed upregulated expression of a series of cytotoxic molecules and chemokines, including *GZMA, GZMH, NKG7* and *CCL4*, while the gene signature of the T_CM_ group included several genes previously reported in studies of advanced-stage CTCL, including *TOX, KIR3DL2, CD40LG, GTSF1, PTTG1, GAPDH, PPIA* and *HSPD1*^27–32^. To eliminate the effect of the cytotoxic nature of CD8^+^ MF and pcALCL on the gene signature of the T_CyEM_ group, a refined analysis including only CD4^+^ MFs in the T_CyEM_ group identified a 19-gene signature. This 19-gene list was highly consistent with the 13-gene signature, including multiple cytotoxic molecules (*GZMA, GZMH* and *NKG7*), which confirmed the cytotoxic phenotype of the malignant T_CyEM_ cells (Supplementary Fig. 3d). Within the gene signature of the T_CM_ group, TOX is a crucial T cell development regulator and has been shown as an adjunctive diagnostic marker in CTCL^28^. KIR3DL2 participates in the regulation of immune response of T cells and has been identified in leukemic CTCL^33,34^. CD40L (encoded by *CD40LG*) expression in MF has been related to increased cell growth and homing of neoplastic cells to the skin^35^. *GTSF1* is one of cancer testis (CT) genes which are aberrantly expressed in CTCL^32^. High expression of GAPDH, indicating a switch to aerobic glycolysis to meet the metabolic demand for tumor proliferation, has been shown to promote aggressive T cell lymphomas^36,37^. *HSPD1* was found to be upregulated in advanced CTCL^38^. *PTTG1* and *PPIA* were described in previous single-cell analyses in CTCL^27,31^.

To better understand the characteristics of the two groups, we evaluated the clinical relevance and functional profiles of malignant T cells in each group (see Methods). Clinically, all five T_CM_ patients were at late stage with large cell transformation (LCT) (Fig. 4a), indicating that patients in this T_CM_ group were related to aggressive clinical courses and poor prognoses^1^. Consistently, malignant T cells in the T_CyEM_ group showed a low exhaustion/high cytotoxicity state, while the T_CM_ group was highly exhausted, supporting the previous finding of high expression levels of exhaustion markers in a portion of CTCL cases^39^, including *PDCD1*, *CTLA4*, *TIGIT*, and *TCF7*, a marker for precursor exhausted T cells^40^ (Fig. 4e and Supplementary Fig. 3e, f). Moreover, discrete activation/proliferation states were identified in different subtypes. Malignant T_CyEM_ cells were highly activated and had low proliferation capacity, while malignant T_CM_ cells were highly proliferative, indicating an increased tendency for progression in this group (Fig. 4f). These phenomena highly recapitulate the features of normal effector memory T cells and central memory T cells^26^.

We further explored the expression patterns of the four meta-programs in the two groups (Supplementary Fig. 3g). In accordance with the two main profiles, malignant T_CyEM_ cells showed higher scores of meta-program 1 and 4 (T cell signaling and activation), while malignant T_CM_ cells exhibited higher scores of meta-program 2 (cell cycle) and meta-program 3 (cell metabolism). To validate this finding, we analyzed the prognostic values of the gene signatures from the two groups in our independent MF cohort. The T_CM_ gene signature was associated with shorter progression-free survival, whereas the T_CyEM_ signature had no significant effect on prognosis, confirming that T_CM_ features were associated with adverse patient outcomes (Fig. 4g and Supplementary Fig. 3h). Therefore, comparing to the T_CyEM_ group, the molecular features of T_CM_ patients were related to more advanced disease stages, aggressive behavior, and adverse prognosis.

These results raise the question of whether T_CyEM_ is a feature restricted to the early stages of MF, so that phenotype switching to T_CM_ occurs when the disease progresses. To address this possibility, we performed trajectory analysis on all CD4^+^ T cells in all samples (Fig. 4h). Starting as a benign naïve T cell subset, malignant T_CyEM_ and T_CM_ cells moved towards opposing divergent branches. Notably, the cellular trajectories of Tregs developing from naïve CD4^+^ T cell were closer to those of the malignant T_CM_ cells. A recent single-cell study identified FOXP3 as a driver of clonal evolution in Sézary cells^31^, which raised the question of whether CTCL with central memory T cell phenotype was Treg origin. However, Tregs did not show apparent CNVs (Fig. 1d) and had no overlapping clonotypes with the malignant cells in the T_CM_ group (data not shown). Therefore, there was no evidence for Treg-derived malignant T cells in our study.

This finding strongly suggests that malignant T_CyEM_ and T_CM_ T cells represented two distinct tumor origins rather than a phenotypic transition in the process of tumor progression. This hypothesis was further validated by patient MF7, a T_CM_ group patient with more than ten years of follow-up assessment. This patient was diagnosed with early-stage MF (T1N1M0B0) ten years ago. The patch/plaque-stage skin lesions obtained from MF7 ten years ago exhibited a T_CM_ phenotype (coexpressing CD45RO and CD27), confirming that the phenotype of the malignant T cells was determined at the outset (Supplementary Fig. 3i).

We also note that TCR-loss clusters were located at the far end of the trajectories of both of the two distinct malignant developmental pathways (Fig. 4h). In each group, the gene expression profiles of TCR-loss clusters were distinct from their TCR-competent counterparts in each group. In particular, the TCR-loss cluster in the T_CyEM_ group expressed even higher levels of T cell activation-related genes in comparison with the TCR-competent cells, including *PTPRC, AHNAK, ITK, ZAP70* and *FYN* (Supplementary Fig. 3j). In the T_CM_ group, increased expression of cellular metabolic genes, including *GAPDH, GTSF1* and *PTTG1* were seen in TCR-loss cluster (Supplementary Fig. 3k). These findings were highly consistent with our NMF analysis, and confirmed that the TCR-loss clusters represent a terminal state of malignant T cells. Loss of TCR expression in CTCL may be a result of clonal evolution to avoid deleterious external stimulation in the process of tumor progression.

Collectively, we established a molecular subtyping scheme in CTCL based on the transcriptome of malignant T cells. We defined a T_CyEM_ group, in which the malignant T cells showed an activated cytotoxic effector memory T cell phenotype, as well as a T_CM_ group, in which malignant T cells were central memory T cell-like cells with high proliferation and exhaustion status. This subtyping may represent the distinct origins of malignant T cells, and are related to patient outcome as assessed in a larger cohort (*n* = 49).

### CD8^+^ TILs are major antitumor effector cells in CTCL and are more exhausted in the T_CM_ group

**Fig. 5:**
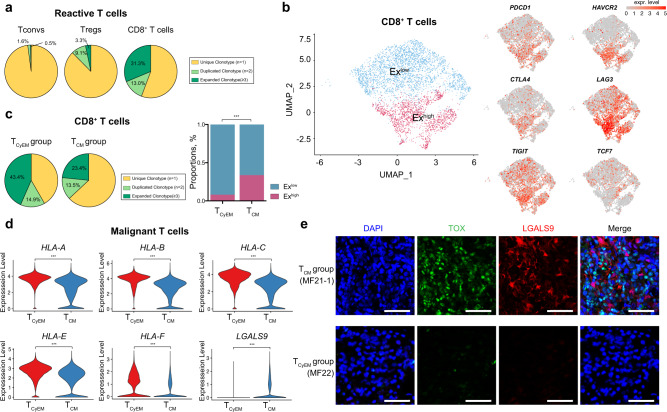
CD8^+^ TILs are major antitumor effector cells in CTCL. **a** Pie charts show the distribution of TCRαβ clonotypes of conventional CD4^+^ T cells (Tconvs), CD4^+^ regulatory T cells (Tregs) and CD8^+^ T cells from the 10× Genomics dataset based on clonal frequency. T cells with TCR clonotype frequency ≥3 are defined as clonally expanded T cells. **b** UMAP plots show all reactive CD8^+^ TILs from the 10× Genomics dataset after re-clustering with the expression of canonical exhaustion markers, including *PDCD1, HAVCR2, CTLA4, LAG3, TIGIT* and *TCF7*. The color scale represents normalized expression. Gray to red: low to high expression. **c** (Left) Pie charts show the distribution of TCRαβ clonotypes of reactive CD8^+^ TILs in the T_CyEM_ and T_CM_ groups based on clonal frequency. T cells with TCR clonotype frequency ≥3 are defined as clonally expanded T cells. (Right) Bar plot shows the proportion of Ex^low^ and Ex^high^ CD8^+^ TILs in the T_CyEM_ and T_CM_ groups. ***p < 0.001. *P* values were calculated using Pearson’s chi-square test. *P* = 2.20 × 10^−16^. **d** Violin plots show the expression levels of MHC-I molecules, including *HLA-A, HLA-B, HLA-C, HLA-E* and *HLA-F*, as well as *LGALS9* in malignant T cells in the T_CyEM_ and T_CM_ groups (log_2_ fold-change >0.5, ***p < 0.001). *P* = 0 (all genes). **e** Representative immunofluorescence staining of TOX (green) and LGALS9 (red) on paraffin-embedded tissue samples from representative T_CyEM_ (upper panel) and T_CM_ (bottom panel) patients. DAPI (blue) was used to visualize cell nuclei. Scale bar = 50 μm. Results are representative of three different samples. Source data for (**c**) are provided in the Source Data file.

Tumor progression is mediated by reciprocal interaction between tumor cells and their surrounding TME^41^. The TME influences cancer cells, and in turn cancer cells have been shown to dictate and modify the surrounding TME^13^. The manner in which differences in malignant T cell phenotypes influence the TME in CTCL lesions is not well understood. To this end, we analyzed and compared cells in the TME from the T_CyEM_ and T_CM_ groups, with a focus on tumor infiltrating lymphocytes (TILs). We first investigated the TCR repertoires of reactive T cells in all samples (Fig. 5a). As expected, the reactive CD8^+^ T cell subset harbored the most frequent clonal expansion, and 44.3% of these cells were clonally expanded, suggesting that CD8^+^ TILs are major anti-tumor effector T cells in CTCL. Tregs exhibited a lower level of clonal expansion (less than 20% cells), while CD4^+^ Tconv cells were least expanded (2.1%) (Fig. 5a), in accordance with previous findings that malignant T cells conferred an impaired antitumor immunity upon normal T helper cells in CTCL^16^.

We re-clustered all CD8^+^ TILs and identified two subsets with distinct exhaustion status (Ex^low^ subset and Ex^high^ subset)., defined by the expression of exhaustion markers *PDCD1*, *HAVCR2*, *CTLA4*, *LAG3*, and *TIGIT* (Fig. 5b). Ex^low^ CD8^+^ TILs exhibited a relatively higher *TCF7* expression and lower *PDCD1*, *HAVCR2*, *CTLA4*, *LAG3* and *TIGIT*, indicative of a precursor exhausted T cell phenotype^40^. Ex^high^ CD8^+^ TILs showed a low *TCF7* expression, with higher levels of the other exhaustion markers, suggesting a terminally differentiated exhausted T cell phenotype. Notably, the T_CyEM_ group harbored more Ex^low^ CD8^+^ TILs with more frequent clonal expansion, whereas the T_CM_ group was enriched with more Ex^high^ CD8^+^ TILs featured by a restricted TCR repertoire (Fig. 5c). CD8^+^ TILs are antigen-specific and require class I major histocompatibility complex (MHC-I) restriction. Therefore, MHC-I expression by malignant T cells was explored. Significant down-regulation of MHC-I molecules was observed in the T_CM_ group, which may impair the immune recognition of CD8^+^ TIL cells in this group (Fig. 5d). Moreover, malignant T cells in the T_CM_ group expressed a higher level of Galectin-9, encoded by *LGALS9*, a canonical ligand of exhaustion marker Tim-3 (encoded by *HAVCR2*) (Fig. 5d, e)^42^. The Galectin-9-Tim-3 interaction was shown to be involved in immune escape by human cancers^42^. Therefore, we proposed that global down-regulation of MHC-I molecules and upregulation of galectin 9 collaboratively contributed to tumor evasion from cytolytic CD8^+^ TIL-mediated antitumor immunity in the T_CM_ group.

### The cell origin of malignant T cells defines TME landscapes

**Fig. 6:**
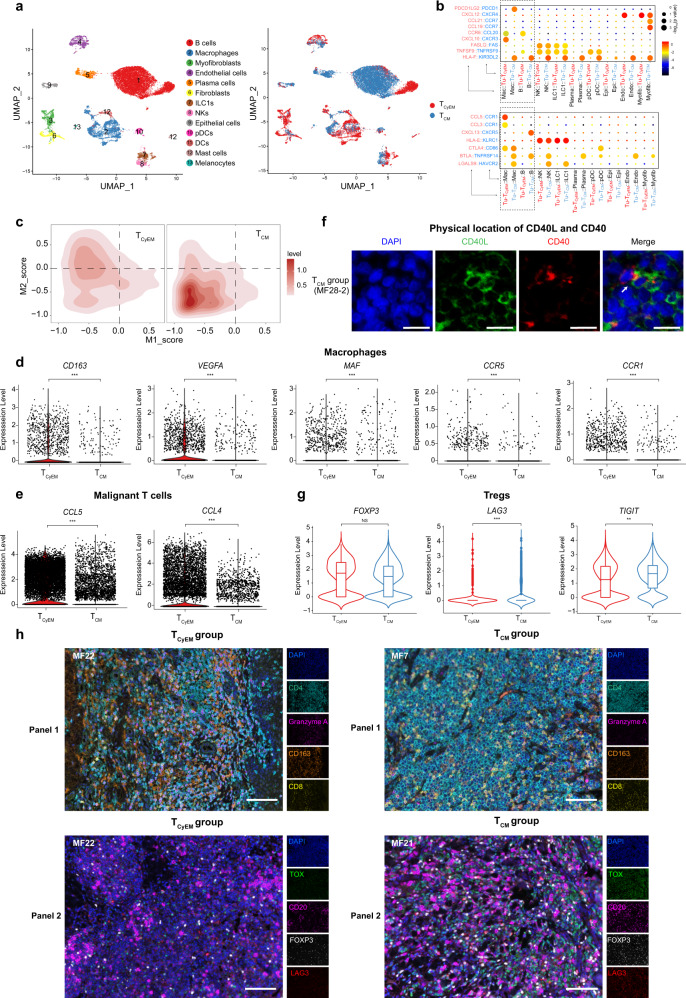
The origin of malignant T cells determines the tumor microenvironment. **a** UMAP plot shows non-T cells from the 10× Genomics dataset. Cells are colored by cell types (left) and molecular subtypes (right). **b** Summary of selected immune-associated ligand-receptor pairs between malignant T cells and the microenvironment in each subtgroup using CellPhoneDB. The size of each dot denotes the *p* value. The color gradient denotes the degree of interaction. Tu-T_CyEM_: malignant T cells in the T_CyEM_ group. Tu-T_CM_: malignant T cells in the T_CM_ group. Mac macrophages, B B cells, Epi epithelial cells, Endo endothelial cells, Myofib myofibroblasts. **c** 2D density plots show the M1 and M2 scores of macrophages in two groups from the 10× Genomics dataset. M1 and M2 score gene lists are provided in Supplementary Table 1. **d** Violin plots show the expression levels of selected genes of macrophages in the T_CyEM_ and T_CM_ groups (log_2_ fold-change >0.5, ***p < 0.001); Two-sided Mann–Whitney *U*-test. *P* = 9.98 × 10^−30^ (*CD163*), 9.24 × 10^−27^ (*VEGFA*), 4.01 × 10^−10^ (*MAF*), 4.17 × 10^−6^ (*CCR5*) and 3.52 × 10^−18^ (*CCR1*). **e** Violin plots show the expression levels of chemokine genes *CCL5* and *CCL4* of malignant T cells in the T_CyEM_ and T_CM_ groups (log_2_ fold-change >1, ***p < 0.001); Two-sided Mann–Whitney *U*-test. *P* = 0 (*CCL5* and *CCL4*). **f** Immunofluorescence staining of CD40LG (green) and CD40 (red) on paraffin-embedded tissue. DAPI (blue) was used to visualize cell nuclei. Scale bar = 20 μm. Results are representative of three different samples. **g** Violin plots show the expression levels of Treg markers in the T_CyEM_ and T_CM_ groups (ns: not significant; ***p < 0.001; **p < 0.01); Two-sided Mann–Whitney *U*-test. *P* = 1 (*FOXP3*), 1.68 × 10^−8^ (*LAG3*) and 3.86 × 10^−3^ (*TIGIT*). **h** Multicolor IHC staining of tumor tissue samples to determine the expression levels of CD4 (green), granzyme A (magenta), CD163 (orange) and CD8 (yellow) in Panel 1 and the expression levels of TOX (green), CD20 (magenta), FOXP3 (white) and LAG3 (red) in Panel 2. DAPI (blue) was used to visualize cell nuclei. Scale bar = 100 μm. Results are representative of three different samples. See also Supplementary Fig. 4g. Source data relating genes in (**a**) and (**c**) are provided in the Source Data file.

Emerging data have highlighted the complexity and heterogeneous composition of the TME in T cell lymphoma^43^. To gain more insight into the potential roles of other immune cells in the TME, we next re-clustered non-T cells from all samples to reveal the global immune landscape (Fig. 6a). Among the diverse cellular components, B cells and macrophages were the dominant cell populations. Interestingly, the T_CyEM_ group showed a relatively higher fraction of macrophages, and the T_CM_ group had remarkably higher abundance of B cells and plasma cells (Fig. 6a and Supplementary Fig. 4a).

A subset of NK cells, an important component of cytotoxic immune cells, existed in all samples. The NK subset displayed a CD16^high^ CD56^low^ (encoded by *FCGR3A* and *NCAM1*, respectively) phenotype, considered as a mature and highly cytotoxic state^44^ (Supplementary Fig. 4a). In addition, a subset of ILC1s exhibiting NK-like gene markers (*NKG7*, *NCR1* and *TBX21*) with an *IL7R*^high^/*EOMES*^*low*^ molecular profile was identified as previously described^45^. NK cells demonstrated cytolytic capacity greater than that of ILC1s, as suggested by the expression levels of cytotoxic markers (Supplementary Fig. 4a). The NK/ILC1 ratios of the T_CM_ and T_CyEM_ groups were comparable, suggesting that NK cells were universally activated in all CTCL cases and may serve as a therapeutic agent for anti-tumor immunotherapy in CTCLs.

Extensive interactions between malignant T cells and various immune cells in both groups were predicted by immune-associated ligand-receptor pairs using the CellPhoneDB repository (see Methods and Fig. 6b). The reciprocal tumor/macrophage and tumor/B cell interaction patterns were distinct between the two groups, prompting us to explore their respective roles in the two CTCL subtypes.

M1 and M2 scores for tumor associated macrophages (TAMs) were calculated according to previously reported markers^46^. Notably, a higher M2 score was observed in TAMs in the T_CyEM_ group (Fig. 6c). *CD163*, a classic M2 macrophage marker, was highly expressed in the T_CyEM_ group (Fig. 6d and Supplementary Fig. 4b, c). The presence of CD163-expressing M2 TAMs has been associated with poor clinical outcomes in CTCL, and depletion of M2 macrophage was found to suppress CTCL development in vivo^10,47,48^. CD163^+^ M2 macrophages could foster an immunosuppressive and pro-tumorigenic inflammatory microenvironment by inducing T cell tolerance and producing pro-angiogenic cytokines such as vascular endothelial growth factor (VEGF)^10,49^. Accordingly, we observed high expression levels of *VEGFA* and *MAF* (encoding a key transcriptional factor of IL10) in the macrophage population in T_CyEM_ patients (Fig. 6d).

To determine the interplay between M2 TAMs and malignant T cells in T_CyEM_ patients, further analysis revealed significantly upregulated *CCL5* and *CCL4* in malignant T cells, coupled with higher expression of their classical receptors, *CCR1* and *CCR5*, in the macrophage cluster (Fig. 6d, e). CCL5 and CCL4 are involved in regulating macrophage trafficking, and CCL5/CCR5 interaction was reported to induce the polarization of CD163^+^ M2 in cancers^50^. Therefore, we proposed that malignant T_CyEM_ cells appeared to produce high levels of CCL5 and CCL4 to recruit and polarize CD163-expressing M2 TAMs, forming a tumor-supporting environment in T_CyEM_ cases.

On the other hand, the B cell cluster, which was highly enriched in T_CM_ patients, expressed a high level of *CD40*, which is associated with B cell activation and Breg differentiation under CD40L stimulation (Supplementary Fig. 4a)^51^. Since *CD40LG* was included in the gene signature of malignant T cells in the T_CM_ group, we hypothesized that malignant T cells may employ the CD40L/CD40 axis to activate B cells in T_CM_ cases. We confirmed the presence of B cells with high tumor infiltration capacity in this group by immunostaining (Supplementary Fig. 4b, c). CD40L-expressing malignant T cells were located in proximity to CD40-expressing B cells in T_CM_ lesions (Fig. 6f, h and Supplementary Fig. 4g). We further validated the close relationship between B cells and T_CM_ cells in our independent MF cohort, and we identified a positive correlation between malignant T_CM_ marker *CD40LG* and mature B cell marker CD20 (encoded by *MS4A1*) (Supplementary Fig. 4d).

B cells are abundant in many human cancers and play cancer-specific roles in antitumor immunity^52^. B cells have been reported to be upregulated in a portion of MF patients^53^. A recent study reported that depletion of mature B cells in an aggressive folliculotropic MF patient achieved improved disease control, suggesting a deleterious effect of B cells in CTCL^11^. Studies have revealed that B cells, especially Bregs, could promote tumor progression by inducing resting CD4^+^ T cells to transform into Tregs in various cancers^51^. Although the T_CM_ and T_CyEM_ groups showed a similar number of Tregs and similar expression levels of *FOXP3*, the Treg population of T_CM_ cases showed higher expression levels of *LAG3* and *TIGIT*, suggesting enhanced suppressor activity in this subset of cells^54,55^(Fig. 6g and Supplementary Fig. 4e, f). Specifically, the LAG3^+^ Treg has been recognized as a subset of cells endowed with potent immunosuppressive capacity, and this type of Treg has been found to preferentially expand in tumors^56^. Therefore, we proposed that malignant T cells activated B cells in T_CM_ patients via a CD40L/CD40 axis, and highly infiltrated B cells may promote tumor progression by activating Tregs.

Taken together, our results demonstrate the roles of M2 macrophages and the B cell-Treg axis in the TMEs of the T_CyEM_ and T_CM_ groups, respectively. To corroborate and illustrate the intricate interactions between malignant T cells and the TME, multicolor IHC staining of tumor tissues was performed. The interplay between M2 macrophages/T_CyEM_ cells and B cells/LAG3^+^ Tregs/T_CM_ cells was demonstrated in the T_CyEM_ and T_CM_ groups (Fig. 6h and Supplementary Fig. 4g). Our results suggest that the diversity of malignant T cell origins determines the heterogeneity of TME, which may contribute to patient outcomes. Thus, anti-CTCL immunotherapy should be tailored according to each patient’s molecular subtype.

## Discussion

High-throughput multi-dimensional single-cell analysis provides a powerful tool to reveal the dynamics of tumor heterogeneity. In our study, using scRNA-seq with paired TCR V(D)J sequencing, we carried out a comprehensive transcriptional analysis on 19 skin lesions from 15 patients across two major CTCL variants MF and pcALCL, and depicted the nature of malignant T cells and their multicellular ecosystems. We put forward a tumor evolution model underlying the pathogenesis of CTCL and present a dimension of molecular subtyping based on tumor origins.

We adopted a reliable approach to precisely distinguish malignant T cells from reactive T cells, providing a solid basis for achieving a deep understanding of the pathobiology of CTCL. Our data lend strong support for a monoclonal origin for malignant T cells derived from mature skin-homing T cells, in contrast with recent studies demonstrating an oligoclonal or polyclonal nature of CTCL^8^. This discrepancy might be caused by contamination of reactive T cells, because laser-capture microdissection based on cell morphology has been widely used to dissect tumor cells^8^. We found that reactive T cells, especially CD8^+^ TILs, displayed a substantial amount of clonal expansion, which could result in the designation of polyclonality in CTCL lesions. To a lesser extent, the absence of allelic exclusion of *TCRA* might be another reason for this discrepancy^14^. The bi-allelic *TCRA* expression has been previously reported on Sézary cells by WES study^57^. Our study was able to pair the bi-allelic *TCRA* to *TCRB* sequence in the single-cell dimension to demonstrate the monoclonal nature of malignant T cells. Therefore, the single-cell approach appears to be a superior method for tracing the development of tumor progression in future studies.

Interestingly, malignant T cells in a portion of patients lacked TCR expression, which was previously reported in Sézary cells^57^. These TCR-loss clusters exist in both T_CyEM_ and T_CM_ groups, representing a universal phenomenon in CTCL, although the clinical significance regarding patient prognosis was not clear due to the limited sample size of our scRNA-seq cohort. Down-regulation of TCR expression represents a negative feedback mechanism for constraining T cell effector function to avoid excess inflammatory damage in reactive T cells^21^. Whether malignant T cells induced a similar mechanism to prevent harmful signal transduction during tumor progression warrants further exploration.

Our data revealed diverse transcriptome programs in the malignant T cells within each sample, demonstrating remarkable subclonal evolution in CTCL. This finding was consistent with a recent study on Sézary syndrome, in which malignant T cells from peripheral blood developed multiple subclones according to culture conditions^58^. We found that distinct activation/proliferation status of the malignant T cells determined the properties of each subclone. Malignant T cells demonstrated either a high proliferation score or a high activation score, suggesting diverse underlying genetic backgrounds. These results are consistent with a previous genomic analysis revealing that putative driver oncogenes in CTCL were varied and involved multiple pathways, including T cell activation, chromatin modification, and cell cycle control^59^. Our prognosis analyses on each meta-program across all samples also demonstrated that the intra-tumor and inter-tumor heterogeneity in the composition of the activation/proliferation cell clusters determined the clinical behavior of each skin lesion and the patient prognosis.

**Fig. 7:**
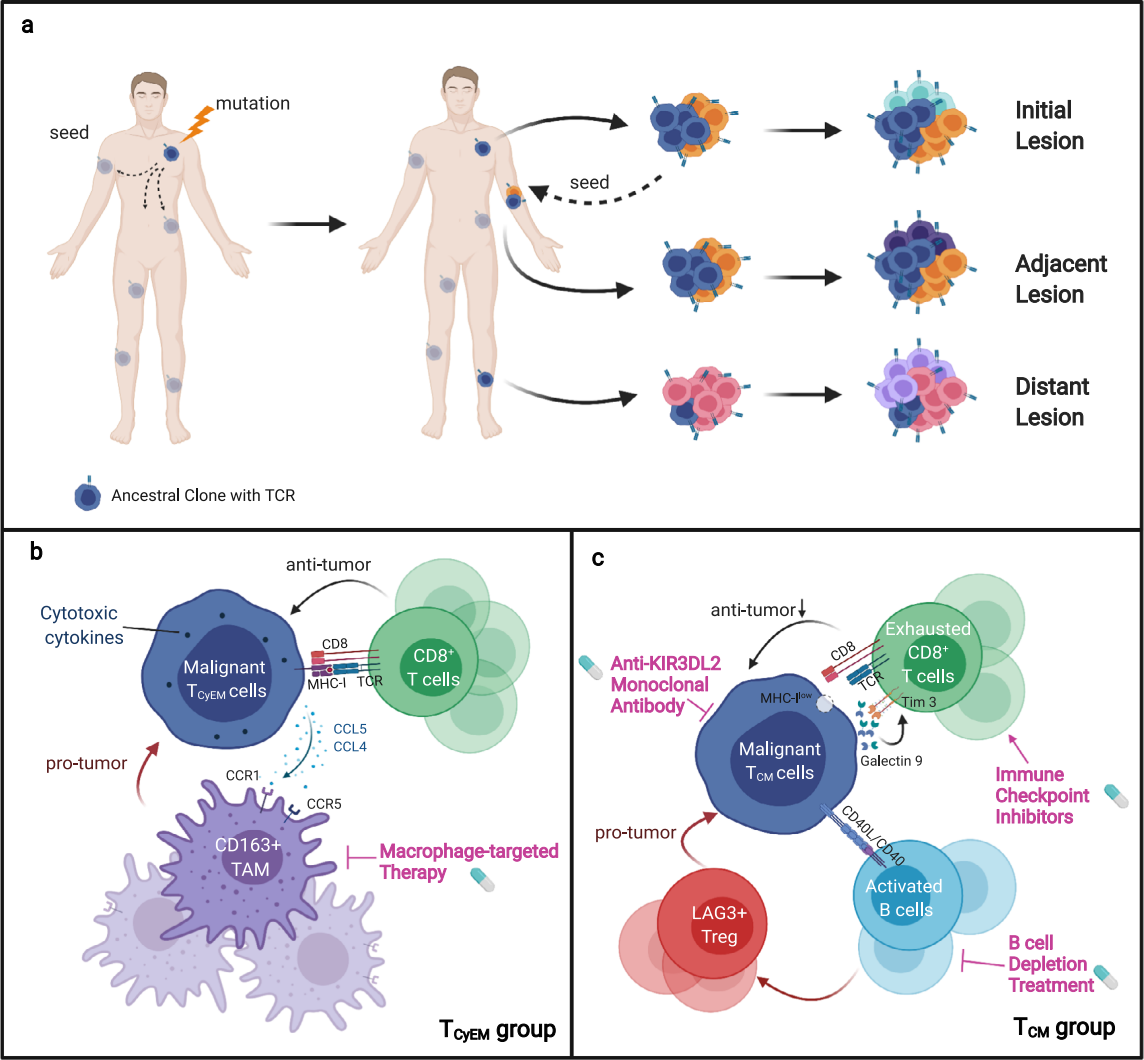
A schematic representation of this study. **a** Single-cell RNA sequencing of cutaneous T cell lymphoma reveals a multi-step seeding model of monoclonal malignant T cells. **b**, **c** A molecular subtyping scheme based on the tumor origins and distinct tumor immune microenvironments, providing insights into therapeutic interventions for CTCL.

Our data confirmed the monoclonal origin of malignant T cells across skin lesions in individual CTCL patients. Because each skin tumor is identifiable once fully established, and accessible to biopsy, we are able to show a multi-step and parallel transformation model for malignant T cells: a single mutated ancestor of the malignant T cell clonally expands and seeds into skin niches at different anatomical sites long before clinically detectable skin lesions occur. Parallel subclonal evolution occurs independently over time and results in the development of new skin rashes. As the skin tumor evolves, multiple waves of dissemination of subclones occur and colonize adjacent anatomical sites, developing new lesions (Fig. 7a). This model explains the intra-lesion heterogeneity and inter-lesion diversity of CTCL patients, as well as differences in the therapeutic vulnerabilities of lesions at different anatomical sites.

In contrast with solid tumors which initiate from a primary site, or the skin involvement of hematological malignancies which demonstrate homogenous skin lesions^60^, CTCL, especially MF, generally begins with multiple skin lesions appearing in succession across long time intervals, even decades, and the lesions on different anatomical sites are varied^61^. Patches, plaques, and tumor-stage lesions may exist simultaneously on the same patient, and they may show distinct responses to treatment^9^. Our findings suggest that skin lesion spreading is an evolutionary process with temporal and spatial dynamics that highly resemble those of the multi-step process of metastatic colonization by solid tumors^24,62^.The seeding events take place across the whole course of the disease development and may depend on the characteristics of malignant T cells and the microenvironment of different skin niches.

The route of malignant T cell dissemination is still debated. It has been reported that skin-resident T-cells do not recirculate; they survive and proliferate in the skin without migrating to the lymph nodes^63,64^. More recent studies have shown that human skin CD4^+^ resident memory T cells can exit the skin, reenter the circulation, and travel to secondary human skin sites^65,66^, supporting our model of the spreading of CTCL lesions. Previous studies with TCR-sequencing also supported this model by demonstrating low but detectable malignant clones in adjacent non-lesional skin and peripheral blood in patients with skin-limited CTCL^67,68^. Noting that our model is based on a limited sampling of skin lesions from a limited number of patients, it is impossible to conclude when the cells first disseminate and whether the temporal or the spatial aspect determines the differences in the trajectories. Factors other than time and anatomical proximity may also play vital roles in the process of tumor evolution. Future large‐scale studies that involve repeated biopsies of multiple skin samples from the same patient combined with longitudinal and spatial sampling will help to corroborate this model. Robust bioinformatic analysis will be required for reliable documentation of malignant T cell dissemination patterns in CTCL.

We proposed a molecular subtyping scheme for CTCL based on the transcriptomic features of malignant T cells. Our findings expand our understanding of CTCL cellular origin of CTCL, which was first elucidated by a seminal study demonstrating that MF arises from a skin-resident effector memory T cell subset, while Sézary syndrome arises from central memory T cells with variable expression of skin addressin CLA^5^. We excluded the possibility that the five patients in the T_CM_ group were Sézary syndrome cases, because none of them were in B2 stage. Therefore, our data showed varied T cell subset origins within MF patients. This finding was supported by a recent study describing discrete CD4^+^ T-cell subsets in the neoplastic cells of MF, including naive T cells (T_N_), T_CM_, T_EM_ and T_EMRA_ subsets^25^. We identified distinct molecular signatures in the T_CM_ and T_CyEM_ subtypes. The T_CyEM_ group showed high expression of cytotoxic molecules. Accordingly, natural cytotoxicity receptor NKp46 and granzyme B have been reported to be expressed by atypical cells in some CD4^+^ CTCL cases, including MF and pcALCL patients^69–71^. We also observed that *CD28* was downregulated in this group (Supplementary Fig. 3a), which was consistent with a previous study demonstrating that loss of CD28 expression was a hallmark of CD4^+^ cytotoxic T cells^72^. This group contains CD4^+^ MF, CD8^+^ MF, and pcALCL cases, suggesting that a portion of MF are closely related to pcALCL. These results may also explain the highly overlapping histology features between pcALCL and MF, and the frequent concurrence of pcALCL and MF in the same patient^39^. The gene signatures of the T_CM_ group include *TOX*, *KIR3DL2* and *GTSF1*, which were previously reported in tumor-stage MF and Sézary syndrome^32,34,73,74^. *TOX* was shown to be highly expressed in both MF and Sézary syndrome, and its expression is related to poor patient prognosis^28,73^. KIR3DL2 has been as a diagnostic marker for Sézary syndrome^34^. KIR3DL2-targeted therapy shows potent antitumor activity against CTCL in preclinical and clinical studies^33^. The T_CM_ group consists of pure CD4^+^ MFs, and its signature is associated with an adverse effect on patient survival, suggesting that some CD4^+^ MF lesions may share a cellular origin with Sézary syndrome and could require more aggressive treatment. Targeted treatment for Sézary syndrome, including anti-KIR3DL2 antibodies, may be applicable in this group of MF patients. Therefore, MF shows a diversified T cell subset origin, which underlies its varied biological and clinical characteristics.

We showed that the origins of malignant T cells determined the particular characteristics of their distinct TMEs (Fig. 7b, c), which could be exploited to develop tailored immunotherapy strategies for different subtypes of patients. Tumor-associated macrophages, especially M2 TAMs, promote angiogenesis, tumor cell invasion, intravasation, and tumor metastasis, and macrophages have been shown to be good targets for anticancer therapy^49^. Bexarotene, an FDA-approved drug for advanced CTCL, has been shown to reduce production of CCL22 from macrophages^75^. Thus, bexarotene, macrophage-targeted therapy, or drugs repolarizing macrophages, e.g. interferon α^76^, may benefit patients in the T_CyEM_ group. Alternatively, malignant T_CM_ cells may evade anti-lymphoma immunity by an orchestrated interaction among malignant T cells, exhausted CD8^+^ TILs, B cells, and Tregs. Administration of immune checkpoint blockers or B cell depletion treatment may show enhanced efficacy in patients in the T_CM_ group. The major obstacle to conducting clinical trials in CTCL patients is that the response rates of almost all available treatments are highly variable. Therefore, molecular subtyping is critical to stratify CTCL patients. Future precision medicine for CTCL patients relies on further elucidation of the distinct characteristics of the tumor ecosystem of each patient subset.

This study, however, is subject to several limitations. First, our study is mainly descriptive, with limited sample size. Further validation studies with a large cohort in CTCL will be required. Second, our sorting strategy in scRNA-seq aimed to maximize the sequencing of T-cell populations; thus, non-T cells were sorted disproportionally in the process of FACS, which may limit the interpretation of the tumor microenvironment analysis. Although we have validated the abundances of the non-T immune cells in each sample, especially macrophages and B cells, with immunohistochemistry, this sorting strategy still influenced the relative number of CD45^-^ non-immune cell population, especially in the Smart-seq samples, which were densely infiltrated by atypical T cells in histology and only a few non-T cells were recovered. Third, due to the technical limitation of scRNA-seq with 10× Genomics platform, the numbers of detected genes from scRNA-seq data are lower than the bulk RNA-seq method^77^. This drawback mainly affects the detection of low-abundance genes, e.g., cytokines and chemokines, limiting the interpretation of T cell functions and cell-cell interactions in this study.

Collectively, our work establishes a conceptual foundation for understanding CTCL tumorigenesis and proposes a molecular subtyping strategy incorporating the phenotypes of malignant T cells and the multicellular immune landscape. Our findings may create therapeutic benefits for CTCL patients and serve as a useful resource for further studies.

## Methods

### Patient sample

Fifteen CTCL patients with informed consent were recruited from the Skin Lymphoma Clinic of Peking University First Hospital in the period from 2018 to 2019. All patients were either treatment naïve or did not receive anti-lymphoma therapy at least 6 months prior to the biopsy. All diagnoses were verified by at least two dermatopathologists according to previously described criteria^9^. For scRNA-seq, thick plaques or tumors were selected and resected from 15 patients corresponding to 19 samples. Each freshly dissociated sample was transported to laboratory immediately. All participants provided written consent for specimen collection and analysis under the study protocol approved by the Peking University First College Hospital Ethics Committee. We have complied with all relevant ethical regulations for work with human participants.

### Tissue dissociation and single-cell suspension preparation

After immediate transportation, each fresh tissue was minced into pieces (<1 mm^3^) on ice and digested with collagenase. To obtain single-cell suspensions with a high viability rate, the solution was transferred into a gentleMACS C Tube (Miltenyi Biotec, 130-093-237) and placed into a gentleMACS octo dissociator (Miltenyi Biotec, 130-095-937), in which it was incubated for 45 min at 37 °C. After digestion, the suspension was filtered using a Falcon 40-μm cell strainer (Corning, 352340). Next, 10 μL cell suspension was used to confirm the cell viability with trypan blue staining (Solarbio, C0040). The sample was then centrifuged and resuspended with serum-free phosphate-buffered saline (PBS) to prepare it for cell staining and flow cytometry.

### Single cell isolation

Each single-cell suspension was stained with CD45-PE and CD3-BV421 for 15 min on ice in the dark. 7-AAD (BD Pharmingen) was added prior to fluorescence-activated cell sorting (FACS). Single cell isolation was performed using FlowJo (v10.7.1, BD Inc, USA). We sorted all T cell populations within the CD45^+^CD3^+^ gate. To elucidate the tumor microenvironment, we also sorted other subtypes disproportionally, including the non-T immune cell population (CD45^+^CD3^−^) and non-immune cell population (CD4^−^). Sorted single cells were collected into 96-well plates for downstream 5′ UMI SmartSeq2-seq (with 10 μL lysis buffer in each well) or mixed into a FACS tube containing 10 μL lysis buffer for downstream drop-based scRNA-seq.

### Immunohistochemistry and immunofluorescence

Paraffin-embedded tissue sections were deparaffinized and rehydrated. After heat antigen retrieval, endogenous peroxidase activity was blocked (only for immunohistochemistry). For immunohistochemistry, the tissue sections were visualized by NDP View2 Viewing software. Staining results were evaluated by Image-Pro Plus 6.0 (Media Cybernetics, USA). For Immunofluorescence, images were captured by fluorescence confocal microscopy (Leica Confocal) and digitalized by Leica software.

Primary antibodies used in immunohistochemistry and immunofluorescence are listed as follows: CD4 (1:100, Abcam, ab133616, clone EPR6855), TCRα (1:100, H-1, santa, sc-515719), CD45RO (1:400, CST, 55618 s, clone UCHL1), CD27 (1:500, Abcam, ab131254, clone EPR8569), granzyme A (1:100, Abcam, ab209205, clone EPR20161), granzyme B (1:800, CST, 17215 s, clone D2H2F), perforin (1:10, eBioscience, 14-9994-82, clone deltaG9), granulysin (1:250, Abcam, ab241333, clone EPR22110-101), TOX (1:300, Abcam, ab237009, clone NAN448B), LGALS9 (1:100, Abcam, ab227046, clone EPR22214), CD40 (1:250, Abcam, ab224639, clone EPR20735), CD40L (1:100, Abcam, ab257319, clone CD40LG/2761), CD20 (1:50, Abcam, ab78237, clone EP459Y), FOXP3 (1:100, Abcam, ab20034, clone 236 A/E7), LAG3 (1:1000, Abcam, ab209236, clone EPR20261), CD163 (1:500, Abcam, ab182422, clone EPR19518), CD8 (1:1000, Proteintech, 66868-1-1 g, clone 1G2B10) and CD45RA (1:300, Millipore, 05-1413, clone MEM 56), TIGIT (1:500, CST, 99567 T, clone E5Y1W).

### Multicolor immunohistochemistry

To evaluate the multicellular ecosystem and the spatial distributions of different cell types within the TMEs of the T_CyEM_ and T_CM_ groups, we performed multicolor immunohistochemistry using FFPE tissue sections of 7 patients from our cohort in this study. The PANO Mutiplex IHC Kit (Panovue) was used for panel 1: TOX (1:200, Abcam, ab237009, clone NAN448B), CD20 (1:50, Abcam, ab78237, clone EP459Y), FOXP3 (1:100, Abcam, ab20034, clone 236 A/E7) and LAG3 (1:100, Abcam, ab209236, clone EPR20261) or Panel 2: GZMA (1:200, Abcam, ab20034, clone EPR20161), CD4 (1:100, Abcam, ab133616, clone EPR6855), CD8 (1:1000, Proteintech, 66868-1-1 g, clone 1G2B10) and CD163 (1:100, Abcam, ab182422, clone EPR19518). The primary antibodies in each panel were sequentially applied, followed by secondary antibody incubation and tyramide signal amplification (TSA). Between each round of staining, antigen retrieval was performed on the slides with heat-treatment. Finally, DAPI was applied to stain nuclei. Each slide was scanned using a Mantra System (PerkinElmer) by capturing the fluorescent spectrum at 20-nm wavelength intervals (420–720 nm) with uniform exposure time, after which the spectra were combined into a single stack image in each panel. The spectrum of each fluorophore and tissue autofluorescence were extracted from the images of single-stained and unstained tissue samples and analyzed by InForm software (PerkinElmer).

### Pre-processing and quality control of scRNA-seq data (10× genomics)

Raw sequencing data from the 10× Genomics platform were converted to fastq format by ‘CellRanger mkfastq’ (v.3.0.2). Next, scRNA-seq reads were aligned to the hg19 reference genome using ‘CellRanger count’ (v3.0.2), and scRNA-TCR data were aligned to the vdj-GRCh38 reference genome. We filtered mitochondrial and ribosomal genes for further analysis. All cellranger output was combined using ‘CellRanger aggr’ (v3.0.2).

To further aggregate and analyze results from the above pipeline using ‘CellRanger aggr’, a stringent data quality control procedure was conducted in the downstream analysis. Only genes detected in at least 10 cells were retained. We filtered out cells with fewer than 500 or more than 5000 detected genes and those with a high mitochondrial content (>10%)^78^.

After discarding poor-quality cells, a total of 58,926 cells were retained for downstream analysis. To normalize the library size effect in each cell, we scaled UMI counts using *scale.factor* = 10,000. Following log transformation of the data, other factors, including “*S.Score*”, “*G2M.Score*”, “*percent.mt*”, “*nCount_RNA*” and “*nFeature_RNA*”, were corrected for variation regression using the *ScaleData* function in Seurat (v3.0.2).

The corrected-normalized data metrics were applied to the standard analysis as described in the Seurat R package. The top 3,000 variable genes were extracted for principal component analysis (PCA). The top 30 principal components were kept for UMAP visualization and clustering. We performed cell clustering using the FindClusters function (*resolution* = 0.2) implemented in the Seurat R package.

### Processing of scRNA-seq data based on 96-well plates

First, raw reads data were demultiplexed based on the cell barcode sequence from Read 2 of the paired-end reads. Then, we trimmed the sequences of read 1 to remove low-quality bases (*N* > 10%), the TSO and polyA tail sequences, and sequences contaminated with adapters. The filtered sequence of read 1 was aligned to the hg19 human genome reference using TopHat (v2.0.12). All the uniquely mapping reads were counted using the htseq-count function from R HTseq package. The duplicated transcripts with identical UMIs in each gene were discarded. Finally, we quantified the transcript number based on the distinct UMIs of each gene in each cell. To visualize the expression matrices of scRNA-seq data based on 96-well plates, the Seurat R package was also applied with a pipeline similar to that described above.

### Cell type annotation and cell state definition

To annotate the cell type for each single cell within all clusters, differential expression genes (DEGs) of each cluster from the 10× Genomics dataset were identified using the FindAllMarkers analysis in the Seurat R package. The top 50 DEGs of each cluster were carefully reviewed. The markers used to define cell types included: *PTPRC*, *CD3E*, *CD3D*, and *CD3G* for T cells; *CD19*, *CD79A*, *MS4A1*, and *CD79B* for B cells; *LYZ*, *CD14*, *CD68*, and *C1QA* for macrophages; *ACTA2*, *TAGLN*, *MYLK*, and *AOC3* for myofibroblasts; *PLVAP*, *CLDN5*, *ENG*, and *AQP1* for endothelial cells; *IGLL5*, *MZB1*, *DERL3*, and *FKBP11* for plasma cells; *DCN*, *COL1A1*, *COL3A1*, and *COL1A2* for fibroblasts; *GNLY*, *KLRB1*, *IL7R*, *GZMB*, *NKG7*, *PRF1*, *FCGR3A*, and *KLRD1* for ILC1s and NKs; *KRT14*, *KRT5*, *KRT6A*, and *KRT17* for epithelial cells; *IRF7*, *LILRA4*, *PLD4*, and *PLAC8* for pDCs; *FSCN1*, *LAMP3*, *CD1E*, and *CCR7* for DCs; *CPA3*, *HPGD*, *CTSG*, and *GATA2* for mast cells; *PMEL*, *TYRP1*, *DCT*, and *MLANA* for melanocytes^46,79^. In parallel, heatmap plots were generated used the top five genes in each cell type. Each cluster from the 5′ UMI scRNA-seq data was annotated following the process described above.

### Cell subclustering analysis

For the subclustering analysis, T cell clusters, non-T cell clusters and CD4^+^ T cells from individual patients (MF21, MF28 and MF30) were extracted from the overall integrated dataset and integrated for further subclustering. After integration, genes were scaled to unit variance. Scaling, PCA, and clustering (using a resolution of 0.6 for T cell clusters, 0.2 for non-T cell clusters and 0.2 for CD4^+^ T cells from individual patients) analyses were performed as described above.

### Copy number variations (CNVs) based on WES Data

We obtained WES data from a subset of samples (*n* = 13) corresponding to 11 patients. DNA was isolated from the CTCL biopsies using the TIANamp genomic DNA Kit (TIANGEN, DP304). Granulocyte DNA isolated from matched blood was used as a germline control sample^80^. We performed paired end, 150 bp read-length sequencing using Illumina NovaSeq 6000 platform. The mean sequencing depths of skin samples and blood control samples were 190.0× (range 101.4× to 256.4×) and 105.9× (range 89.9× to 132.2×), respectively (Supplementary Table 2). For the WES data, the sequenza R package (v3.0.0) was used to call CNVs^81^. Briefly, we used BAM files from the WES data for tumors and matched blood samples as input to calculate the depth ratio, which was normalized based on the GC content bias and data ratio. To acquire segmented copy numbers and estimate tumor purity, the following parameters were used: breaks.method = ‘full’, gamma = 40, kmin = 5, gamma.pcf = 200, kmin.pcf = 200 and assembly = “hg38”. For each tumor sample, the copy numbers of segments were then divided by ploidy following log_2_ transformation. The tumor purity of each sample was in the range of 0.21–0.73 (Supplementary Table 2).

### Inferring CNVs based on scRNA-seq data

To differentiate malignant T cells from reactive T cells, large-scale CNVs were inferred from the scRNA-seq data using the R infercnv package (v1.7.1). Reactive T cells and other cell types were used as controls for the CNV analysis. We estimated the CNV patterns of all cells in each sample using default parameters^12^. For the WES data, the CNV patterns were called by the sequenza R package (v3.0.0) as described above. The true CNV was used as a positive control to identify differences in the two CNV patterns. Malignant T cells were distinguished from reactive T cells mainly based on inferred large-scale CNVs, aneuploid status, feature marker gene expression and cell cluster distribution.

### Signature score analysis based on gene sets

We used a core gene set to perform cell cycle analysis based on 43 G1/S genes and 54 G2/M genes^12^. A cell cycle score was calculated for each cell using the CellCycleScoring R function. Finally, each cell was assigned with a prediction classification in either the S, G2M or G1 phase based on its S and G2M scores.

To evaluate the status of T cell clusters, we evaluated cell states using datasets described in Jin et al. ^12^ and Gene Ontology (GO): a cytotoxicity gene set (*GZMA, GZMB, GZMK, IFNG, NKG7, PRF1, CST7* and *CCL4*), an exhaustion gene set (*PDCD1, LAG3, TIGIT, HAVCR2* and *CTLA4*) and an activation gene set (GO:0002286). We calculated the gene signature scores for these gene sets across all subgroups and samples using the R ggplot2 package (v3.3.2).

### Pseudotime analysis

To construct developmental trajectories along pseudotime for CD4^+^ T cells, including malignant T cells and reactive T cells, the monocle R package (v2.16.0) was applied to the transcriptional expression patterns of the cell subtypes in all samples and individual patients (MF21, MF28 and MF30) from the 10× Genomics dataset. The expression matrices of the scRNA-seq data were analyzed with default settings following the vignette of Monocle 2. We plotted CD4^+^ reactive T cells and malignant cells along the inferred cell trajectories.

### Expression programs of intratumor heterogeneity

To reveal the intratumor heterogeneity of CTCL, a non-negative matrix factorization algorithm documented by the R NMF package (v0.23.0) was applied to malignant T cells from each patient (including the 5′ UMI and 10× Genomics datasets)^82^. For the expression matrix, we filtered genes with standard deviation of expression <0.5 within each patient. NMF was applied to the relative expression values, with all negative values replaced by zero. We successfully extracted five potential programs from each patient, which generated 65 intratumor expression programs among the 13 patients. To investigate common programs among the 65 signatures, the programs were clustered into meta-programs by hierarchical clustering, using overlapping genes (among the 50 top-scoring genes of each program) as a similarity metric. Four meta-programs (two T cell signaling and activation, one cell cycle and one cell metabolism) were identified and further analyzed. For meta-programs, all genes within the programs were defined as the meta-signature. The gene lists of the 4 meta-programs are provided in Source Data file.

### Cell-cell interaction analysis

We conducted cell-cell interactions analysis utilizing the cellphonedb function curated by the CellPhoneDB v.2.0 database (www.cellphonedb.org)^83^. ScRNA-seq counts files and cell type markers were used as input data. According to the expression of a receptor by one cell type and a ligand by another cell type, enriched ligand-receptor interactions between pairs of cell types were calculated. The *P* value for the likelihood of cell-type specificity of a given ligand-receptor complex was calculated based on the proportion of means that were as high as or higher than the actual mean. The significant cell-cell interactions were selected with a *P* value threshold of <0.05. We used the R ggplot2 package (v3.3.2) to visualize the results.

### Mutation calling with WES data

For the WES data, somatic variants were detected using the GATK (v4.1.8.1) Best Practices Pipeline (GATK broadinstitute.org). Paired-end reads were aligned to human genome hg38 (UCSC) using the Burrows-Wheeler Aligner (BWA) (v0.7.17) with default parameters^84^. Samtools (v0.1.19) was used to convert SAM files to compressed BAM files and sort the BAM files by chromosomal coordinates^85^. Then, GAKT MarkDuplicates was used to mark PCR duplicates. Next, base quality recalibration was performed with GAKT BaseRecalibrator and ApplyBQSR. Granulocyte DNA isolated from matched blood was used as a germline control sample^80^. We used GAKT Mutect2 to call somatic SNVs and indels, after which we used GATK FilterMutectCalls to filter out false callings.

### Phylogenetic tree analysis

To reveal the clonal relationship between paired tumors from patients MF21 and MF30, phylogenetic trees were constructed using MEGA-X (v10.2.2)^86^. Sequences 20 bp in length surrounding the non-synonymous mutations (including SNVs and INDELs) were extracted to construct the phylogenetic trees of each patient based on the maximum-parsimony algorithm. All phylogenetic trees were further optimized using Adobe Illustrator. We labeled potential driver events for each patient on each tree.

### Quantification and statistical analysis

In this study, R (v 4.0.3) and GraphPad Prism (v6.1) software were used to conduct statistical analysis. The Mann–Whitney *U* test, Spearman rank correlation test, log-rank test and Pearson’s chi-square test were used. Detailed statistical analysis methods are described in the Figure Legends or main text.

### Illustration tool

Some of the elements of Figs.1a, 3a, d, g and 7a–c were created with BioRender.com (https://biorender.com).

### Reporting summary

Further information on research design is available in the Nature Research Reporting Summary linked to this article.

## Supplementary information

Supplementary Information

Supplementary Data File 1

Supplementary Data File 2

Supplementary Data File 3

Reporting Summary

## Data Availability

All relevant data are available in publicly accessible repositories. The raw data from the scRNA-seq, TCR-seq, bulk exome sequencing and some bulk RNA-seq data have been deposited in the Genome Sequence Archive for human (GSA-Human) under accession number HRA000166. The remaining raw bulk RNA-seq data have been deposited in the Gene Expression Omnibus database under accession numbers GSE168508 and GSE109620^23,87^. To comply with the “Guidance of the Ministry of Science and Technology (MOST) for the Review and Approval of Human Genetic Resources”, we are required to deposit the genomic data of Chinese patients under a controlled access at the GSA in Beijing Institute of Genomics Data Center. To gain access to the raw data under accession number HRA000166, please submit requests to the GSA-Human online page for this study [https://ngdc.cncb.ac.cn]. For scientific research purposes, the access will be granted and the data can be downloaded in a typical one month time window. The remaining data are available within the Article, Supplementary Information or Source Data file. The source data underlying Fig. 1c, g, 2e, g, 3j, k, 4d, 5c, 6a, c and Supplementary Fig. 3d are provided in the Source Data file. Source data are provided with this paper.

## Source data

Source Data File

## Code availability

The computer code for marker selection, clustering, and differential expression is available at https://github.com/husimeng0717/scRNAseq-for-CTCL.
