# Supplementary material for: Single-cell transcriptomics links malignant T cells to the tumor immune landscape in cutaneous T cell lymphoma
Source: Nat Commun. 2022 Mar 3;13:1158. doi: 10.1038/s41467-022-28799-3 (PMC8894386; doi:10.1038/s41467-022-28799-3)
Supplement: Supplementary file 1 — Supplementary Information [file 41467_2022_28799_MOESM1_ESM.pdf]

# Supplementary Figures and Tables

## Single-cell transcriptomics links malignant T cells to the tumor immune landscape in cutaneous T cell lymphoma

Xiangjun Liu[1,2,3,13], Shanzhao Jin[4,5,13], Simeng Hu[4,6,13], Ruoyan Li[4,7], Haihao Pan[1,2,3], Yi Liu[4], Pan Lai[1,2,3], Deshu Xu[4], Jingru Sun[1,2,3], Ziyang Liu[4], Yumei Gao[1,2,3], Yifan Zhao[4], Fengjie Liu[1,2,3], Yu Xiao[1,2,3], Yingyi Li[1,2,3], Yujie Wen[1,2,3], Zhuojing Chen[1,2,3], Bufang Xu[1,2,3], Yuchieh Lin[1,2,3], Menglong Ran[1,2,3], Qianxi Li[1,2,3], Shuxia Yang[1,2,3], Hang Li[1,2,3], Ping Tu[1,2,3], Muzlifah Haniffa[7,8,9], Sarah A. Teichmann[7,10], Fan Bai[4,11,12*], Yang Wang[1,2,3*]

[1]Department of Dermatology and Venerology, Peking University First Hospital, Beijing 100034, China.
[2]Beijing Key Laboratory of Molecular Diagnosis on Dermatoses, Beijing 100034, China.
[3]National Clinical Research Center for Skin and Immune Diseases, Beijing 100034, China.
[4]Biomedical Pioneering Innovation Center (BIOPIC), and School of Life Sciences, Peking University, Beijing 100871, China.
[5]BioMap (Beijing) Intelligence Technology Limited, Block C Information Center Haidian District, Beijing, 100086, China
[6]Academy for Advanced Interdisciplinary Studies (AAIS), and Peking University–Tsinghua University–National Institute of Biological Sciences Joint Graduate Program (PTN), Peking University, Beijing 100871, China
[7]Wellcome Sanger Institute, Wellcome Genome Campus, Hinxton, Cambridge CB10 1SA, UK.
[8]Biosciences Institute, Newcastle University, Newcastle upon Tyne NE2 4HH, UK.
[9]Department of Dermatology and NIHR Newcastle Biomedical Research Centre, Newcastle Hospitals NHS Foundation Trust, Newcastle upon Tyne NE2 4LP, UK.
[10]Theory of Condensed Matter Group, Cavendish Laboratory/Department of Physics, University of Cambridge, Cambridge CB3 0HE, UK.
[11]Beijing Advanced Innovation Center for Genomics (ICG), Peking University, Beijing, China
[12]Center for Translational Cancer Research, First Hospital, Peking University, Beijing 100871, China.
[13]These authors contributed equally to this manuscript.

*Address correspondence to: Yang Wang (yangwang_dr@bjmu.edu.cn) and Fan Bai (fbai@pku.edu.cn)

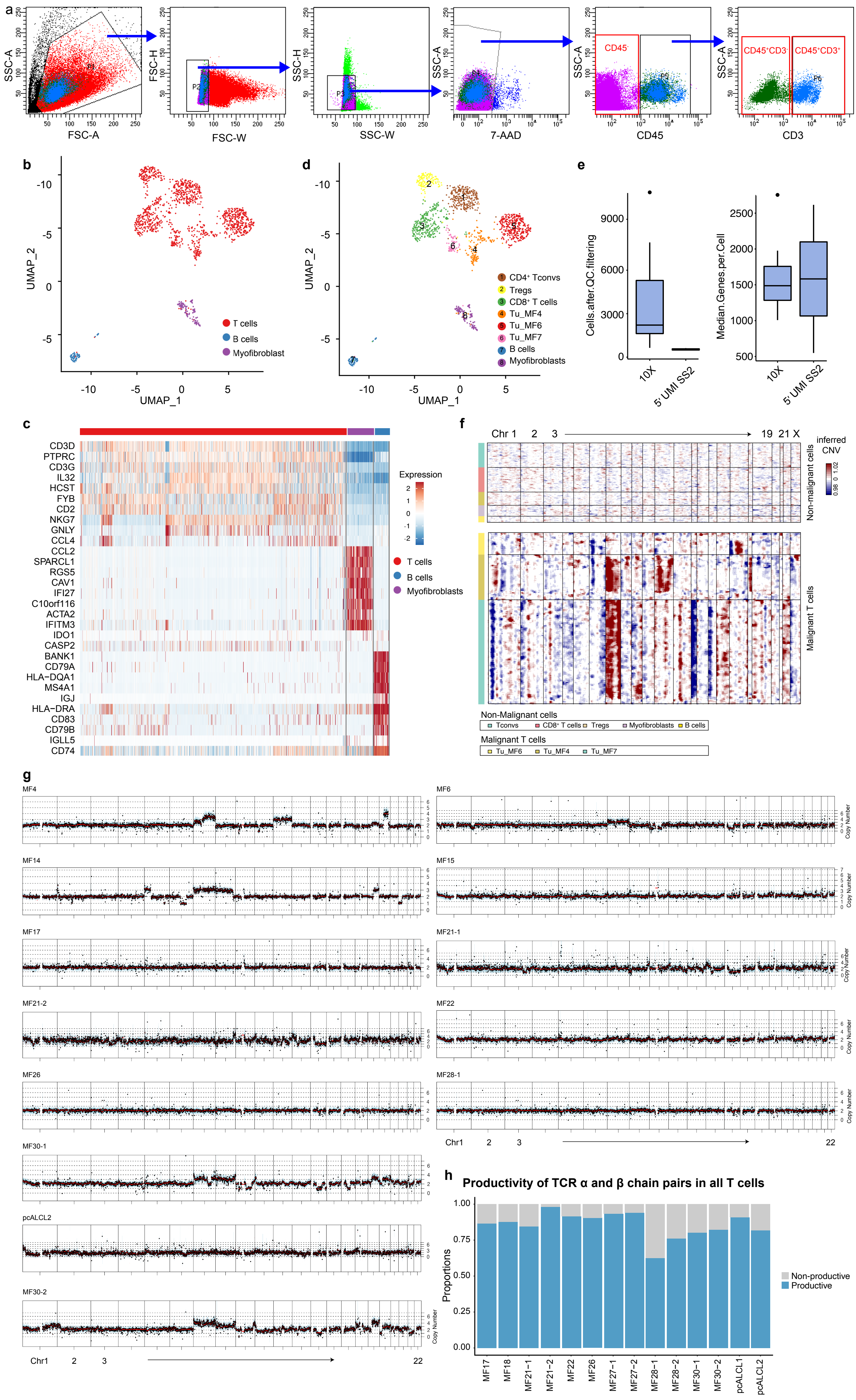

a
SSC-A
FSC-A
FSC-H
FSC-W
SSC-H
SSC-W
7-AAD
CD45⁻
CD45
CD45⁺CD3⁻
CD45⁺CD3⁺
CD3
b
UMAP_2
UMAP_1
T cells
B cells
Myofibroblast
d
CD4⁺ Tconvs
Tregs
CD8⁺ T cells
Tu_MF4
Tu_MF6
Tu_MF7
B cells
Myofibroblasts
e
Cells.after.QC.filtering
Median.Genes.per.Cell
10X
5'UMI SS2
c
CD3D
PTPRC
CD3G
IL32
HCST
FYB
CD2
NKG7
GNLY
CCL4
CCL2
SPARCL1
RGS5
CAV1
IFI27
C10orf116
ACTA2
IFITM3
IDO1
CASP2
BANK1
CD79A
HLA−DQA1
MS4A1
IGJ
HLA−DRA
CD83
CD79B
IGLL5
CD74
Expression
T cells
B cells
Myofibroblasts
f
Chr 1 2 3 19 21 X
inferred CNV
Non-malignant cells
Malignant T cells
Non-Malignant cells
Tconvs
CD8⁺ T cells
Tregs
Myofibroblasts
B cells
Malignant T cells
Tu_MF6
Tu_MF4
Tu_MF7
g
MF4
MF6
MF14
MF15
MF17
MF21-1
MF21-2
MF22
MF26
MF28-1
MF30-1
pcALCL2
MF30-2
Copy Number
Chr1 2 3 22
h
Productivity of TCR α and β chain pairs in all T cells
Proportions
Non-productive
Productive
MF17
MF18
MF21−1
MF21−2
MF22
MF26
MF27−1
MF27−2
MF28−1
MF28−2
MF30−1
MF30−2
pcALCL1
pcALCL2

**Supplementary Figure 1. Single-cell transcriptional profiles of all cells from the 5′ UMI Smart-Seq2 dataset.**

**(a)** FACS gating strategy of $CD45^-$, $CD45^+CD3^-$ and $CD45^+CD3^+$ subsets from skin lesions of CTCL patients, related to Fig. 1a.

**(b and c)** UMAP plot shows 1, 398 high-quality cells in the 5′ UMI Smart-Seq2 dataset. Three cell types were defined by cell-specific markers as described previously, with each cell colored by cell type as annotated (**b**), and malignant T cells were further identified (**c**).

**(d)** Heatmap shows the expression of the top 10 signature genes in each cell type from the 5′ UMI Smart-Seq2 dataset. The expression level was measured as the z-score normalized $\log_2$ level (count+1).

**(e)** Boxplot shows the comparison of the detected cell numbers per sample (left) and the median gene numbers per cell (right) between the 10× Genomics (a total of 58926 cells × 24864 genes) and 5′ UMI Smart-Seq2 (a total of 24864 cells × 4733 genes) datasets after quality control filtering. Boxes represent median and interquartile ranges (IQRs), whiskers are 1.5 × IQR, and dots represent subjects outside the IQR range.

**(f)** Large-scale CNVs of single cells from all samples. CNVs were inferred from the 5′ UMI Smart-Seq2 dataset.

**(g)** The CNV patterns of 13 samples inferred from whole exome sequencing.

**(h)** The productivity of TCR α and β chain pairs in all T cells of each sample.

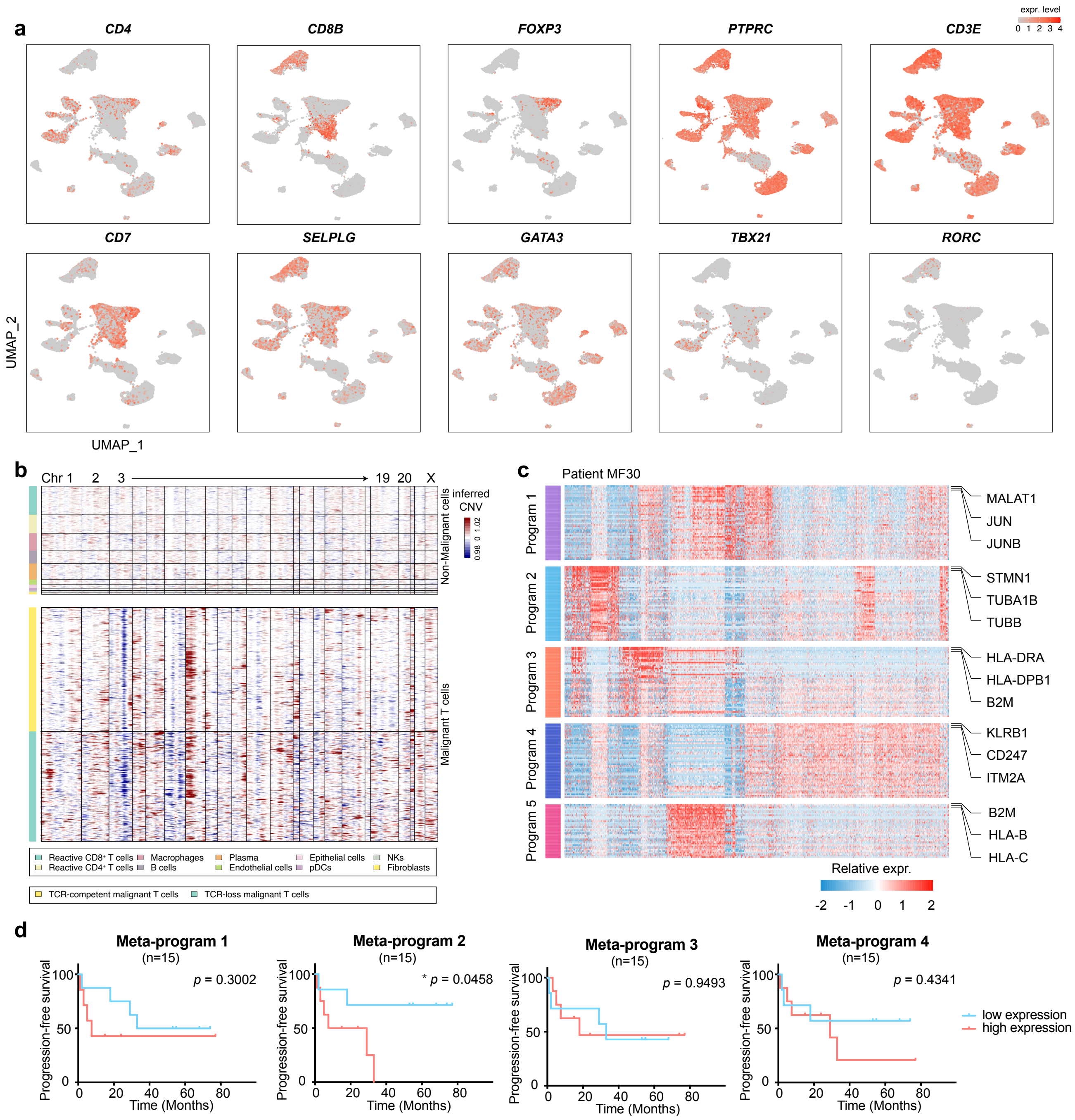
a
CD4
CD8B
FOXP3
PTPRC
CD3E
expr. level
0 1 2 3 4
CD7
SELPLG
GATA3
TBX21
RORC
UMAP_2
UMAP_1
b
Chr 1 2 3 19 20 X
inferred CNV
0.98 0 1.02
Non-Malignant cells
Malignant T cells
Reactive CD8⁺ T cells
Reactive CD4⁺ T cells
Macrophages
B cells
Plasma
Endothelial cells
Epithelial cells
pDCs
NKs
Fibroblasts
TCR-competent malignant T cells
TCR-loss malignant T cells
c
Patient MF30
Program 1
MALAT1
JUN
JUNB
Program 2
STMN1
TUBA1B
TUBB
Program 3
HLA-DRA
HLA-DPB1
B2M
Program 4
KLRB1
CD247
ITM2A
Program 5
B2M
HLA-B
HLA-C
Relative expr.
-2 -1 0 1 2
d
Meta-program 1
(n=15)
p = 0.3002
Meta-program 2
(n=15)
* p = 0.0458
Meta-program 3
(n=15)
p = 0.9493
Meta-program 4
(n=15)
p = 0.4341
Progression-free survival
Time (Months)
low expression
high expression

**Supplementary Figure 2. Intra-tumor heterogeneous transcriptional properties of malignant T cells.**

**(a)** UMAP plot shows the expression of canonical T cell markers in all T cells from the 10× Genomics dataset. The color scale represents normalized expression. Gray to red: low to high expression.

**(b)** Large-scale CNVs of single cells from sample MF28-1. CNVs were inferred from the transcriptome.

**(c)** Heatmap shows five gene expression programs deciphered from a representative patient (MF30) using NMF.

**(d)** PFS analysis of an independent cohort of 15 pcALCL patients. Patients were stratified into low and high expression groups according to median values of scores corresponding to the gene signatures of four meta-programs. *P*-values were calculated using the log-rank test.

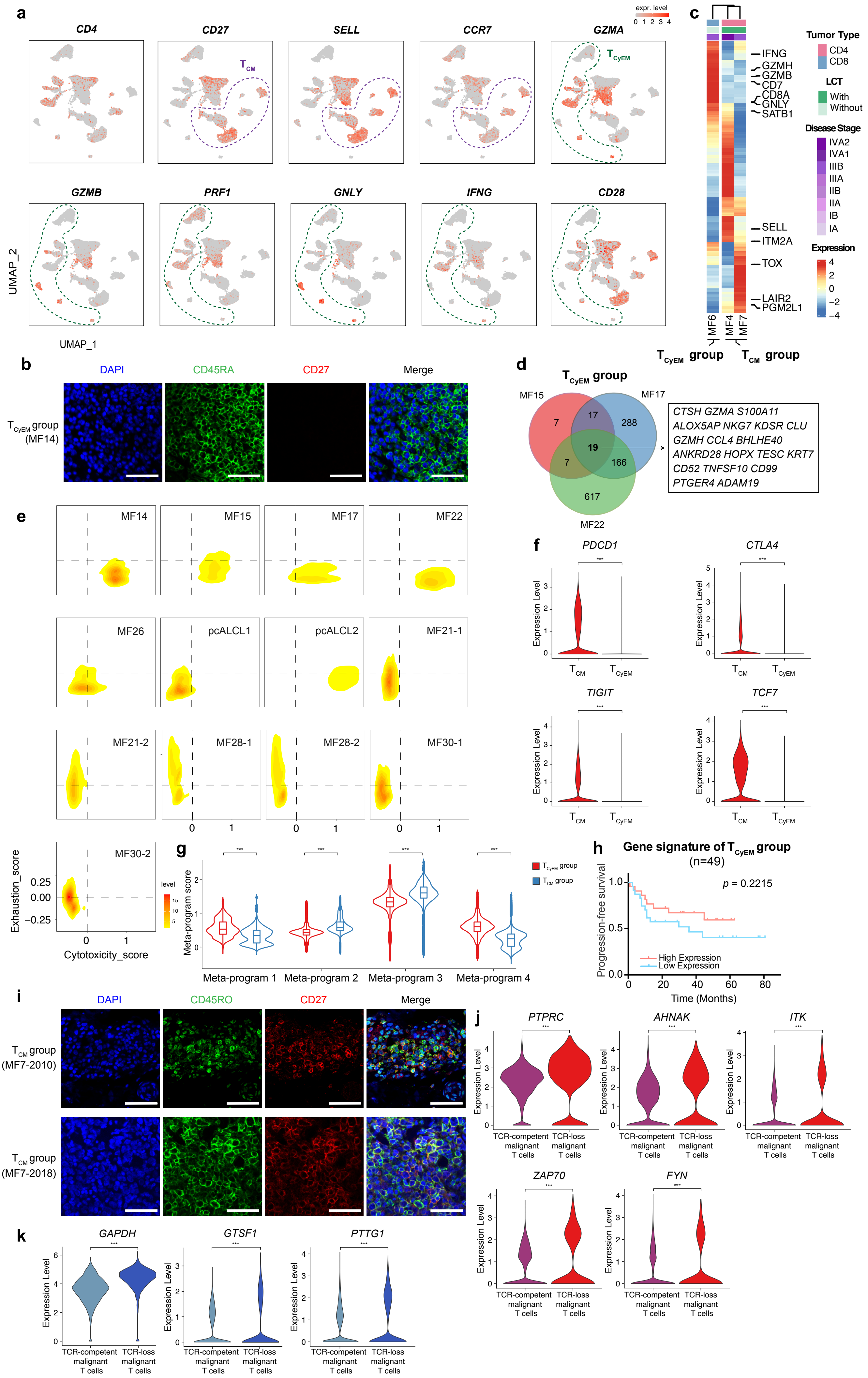
a
CD4
CD27
SELL
CCR7
GZMA
expr. level
0 1 2 3 4
T CM
T CyEM
GZMB
PRF1
GNLY
IFNG
CD28
UMAP_2
UMAP_1
c
IFNG
GZMH
GZMB
CD7
CD8A
GNLY
SATB1
SELL
ITM2A
TOX
LAIR2
PGM2L1
MF6
MF4
MF7
T CyEM group
T CM group
Tumor Type
CD4
CD8
LCT
With
Without
Disease Stage
IVA2
IVA1
IIIB
IIIA
IIB
IIA
IB
IA
Expression
4
2
0
−2
−4
b
DAPI
CD45RA
CD27
Merge
T CyEM group (MF14)
d
T CyEM group
MF15
MF17
MF22
7
17
288
19
7
166
617
CTSH GZMA S100A11
ALOX5AP NKG7 KDSR CLU
GZMH CCL4 BHLHE40
ANKRD28 HOPX TESC KRT7
CD52 TNFSF10 CD99
PTGER4 ADAM19
e
MF14
MF15
MF17
MF22
MF26
pcALCL1
pcALCL2
MF21-1
MF21-2
MF28-1
MF28-2
MF30-1
MF30-2
Exhaustion_score
Cytotoxicity_score
0.25
0.00
−0.25
0
1
level
15
10
5
f
PDCD1
CTLA4
TIGIT
TCF7
Expression Level
T CM
T CyEM
g
Meta-program score
T CyEM group
T CM group
Meta-program 1
Meta-program 2
Meta-program 3
Meta-program 4
h
Gene signature of T CyEM group
(n=49)
p = 0.2215
Progression-free survival
High Expression
Low Expression
Time (Months)
0
20
40
60
80
i
DAPI
CD45RO
CD27
Merge
T CM group (MF7-2010)
T CM group (MF7-2018)
j
PTPRC
AHNAK
ITK
ZAP70
FYN
Expression Level
TCR-competent malignant T cells
TCR-loss malignant T cells
k
GAPDH
GTSF1
PTTG1
Expression Level
TCR-competent malignant T cells
TCR-loss malignant T cells

**Supplementary Figure 3. Molecular subtyping with malignant T cell-intrinsic features.**

**(a)** UMAP plots show the gene expression patterns of all T cells from the 10× Genomics dataset. Two subgroups identified in Fig. 4A are highlighted by dashed circles. The color scale represents normalized expression. Gray to red: low to high expression.

**(b)** Immunofluorescence staining of CD45RA (green) and CD27 (red) on paraffin-embedded tissue of patient MF14. DAPI (blue) was used to visualize cell nuclei. Scale bar = 50 µm. Results are representative of three different samples.

**(c)** Heatmap of unsupervised hierarchical clustering shows the average expression of the DEGs between malignant T cells and their respective reactive $CD4^+$ or $CD8^+$ T cells in each sample from 3 patients in the 5′ UMI Smart-Seq2 dataset ($\log_2$ fold-change >1.5, $p < 0.05$). The bars above the heatmap show the tumor type, LCT information and disease stage. LCT, large cell transformation.

**(d)** Venn diagram illustrates a 19-gene signature from $CD4^+$ MF patients in the $T_{CyEM}$ group.

**(e)** 2D density plots show the cytotoxicity score and exhaustion score of malignant T cells in each sample from the 10× Genomics dataset.

**(f)** Violin plots show that malignant $T_{CM}$ cells expressed a higher level of *PDCD1*, *CTLA4*, *TIGIT,* and *TCF7* in comparison with malignant $T_{CyEM}$ cells; Two-sided Mann-Whitney U-test. $^{***}p < 0.001$. $P = 0$ (all genes).

**(g)** Violin plots show the scores of the four meta-programs in the $T_{CyEM}$ and $T_{CM}$ group; Two-sided Mann-Whitney U-test. $P \leq 2.00 \times 10^{-16}$ in the four meta-programs.

**(h)** PFS analysis of an independent cohort of 49 tumor-stage MF patients. Patients were stratified into low expression and high expression groups according to median values of scores corresponding to the 19-gene signature of the $T_{CyEM}$ groups identified in Supplementary Fig. 4d. *P*-values were calculated using the log-rank test.

**(i)** Immunofluorescence staining of CD45RO (green) and CD27 (red) on thin plaque tissue isolated in 2010 (upper) and tumor tissue isolated in 2018 (lower) from patient MF7. DAPI (blue) was used to visualize cell nuclei. Scale bar = 50 µm. Results are representative of three different samples.

**(j)** Violin plots show that TCR-loss malignant T cells in the $T_{CyEM}$ group express higher levels of T cell activation-related genes in comparison with TCR-competent malignant T cells ($\log_2$ fold-change >0.14, $^{***}p < 0.001$); Two-sided Mann-Whitney U-test. $P = 1.11 \times 10^{-10}$ (*PTPRC*), $4.68 \times 10^{-4}$ (*AHNAK*), $2.82 \times 10^{-7}$ (*ITK*), $6.73 \times 10^{-7}$ (*ZAP70*) and $8.28 \times 10^{-4}$ (*FYN*).

**(k)** Violin plots show that TCR-loss malignant T cells in the $T_{CM}$ group express higher levels of *GAPDH, GTSF1* and *PTTG1* in comparison with TCR-competent malignant T cells ($\log_2$ fold change >0.15, $^{***}p < 0.001$); Two-sided Mann-Whitney U-test. $P = 2.20 \times 10^{-16}$ (*GAPDH*), $1.60 \times 10^{-5}$ (*GTSF1*) and $2.20 \times 10^{-16}$ (*PTTG1*).

Source data for **(d)** are provided in the Source Data file

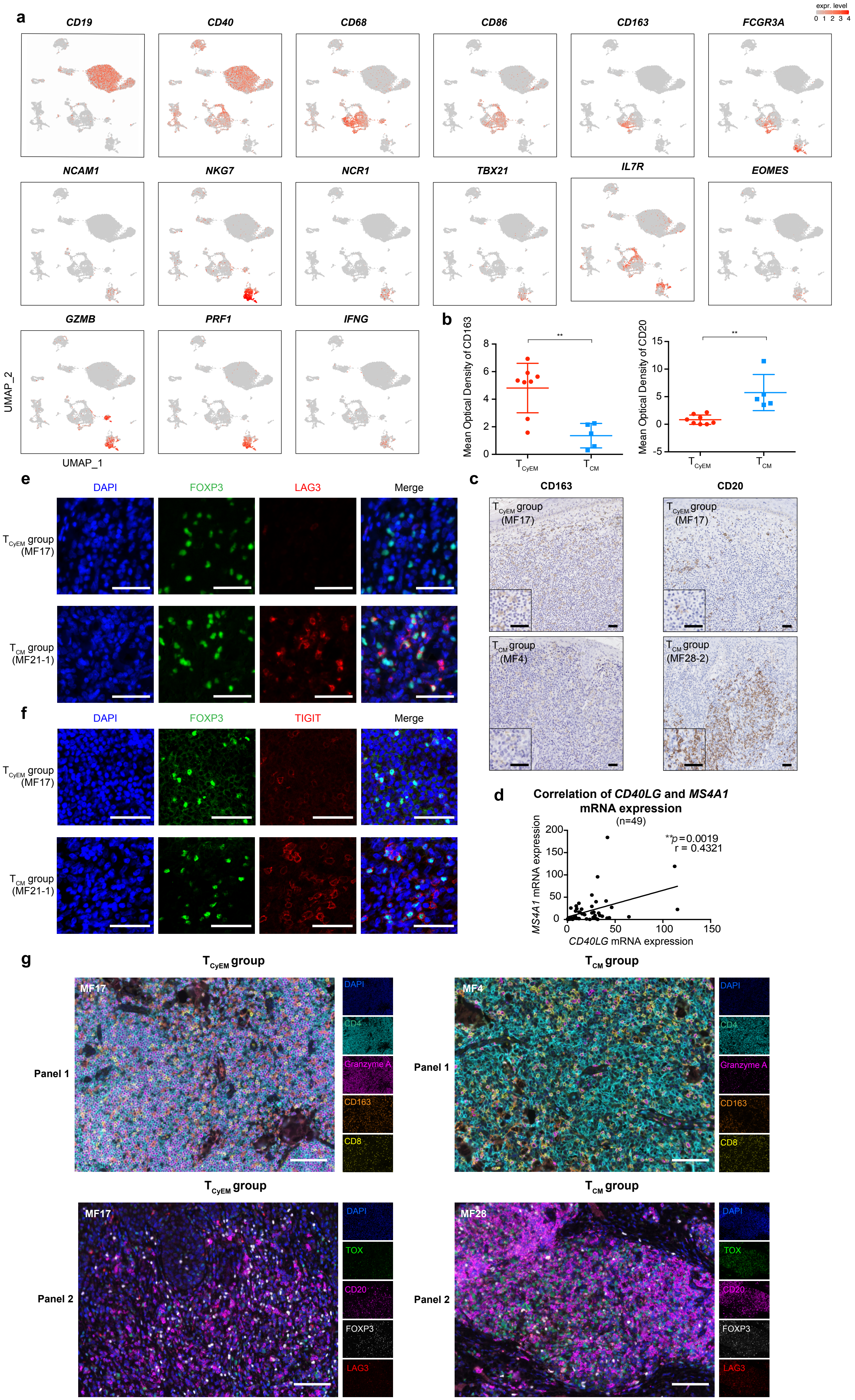
a
CD19
CD40
CD68
CD86
CD163
FCGR3A
expr. level
0 1 2 3 4
NCAM1
NKG7
NCR1
TBX21
IL7R
EOMES
GZMB
PRF1
IFNG
UMAP_2
UMAP_1
b
Mean Optical Density of CD163
Mean Optical Density of CD20
**
$T_{CyEM}$
$T_{CM}$
c
CD163
CD20
$T_{CyEM}$ group (MF17)
$T_{CM}$ group (MF4)
$T_{CM}$ group (MF28-2)
d
Correlation of CD40LG and MS4A1 mRNA expression
(n=49)
**p=0.0019
r = 0.4321
MS4A1 mRNA expression
CD40LG mRNA expression
e
DAPI
FOXP3
LAG3
Merge
$T_{CyEM}$ group (MF17)
$T_{CM}$ group (MF21-1)
f
DAPI
FOXP3
TIGIT
Merge
$T_{CyEM}$ group (MF17)
$T_{CM}$ group (MF21-1)
g
$T_{CyEM}$ group
$T_{CM}$ group
Panel 1
Panel 2
MF17
MF4
MF28
DAPI
CD4
Granzyme A
CD163
CD8
TOX
CD20
FOXP3
LAG3

**Supplementary Figure 4. The interaction between malignant T cells and the TME in each subtype.**

**(a)** UMAP plot shows the expression of canonical cell type markers in non-T cells from the 10× Genomics dataset. The color scale represents normalized expression. Gray to red: low to high expression.

**(b)** The mean optical density of CD163 and CD20 in the $T_{CyEM}$ (n=8) and $T_{CM}$ (n=5) groups. Data are presented as median ± interquartile range. Two-sided Mann-Whitney U-test, $**p < 0.01$. Each experiment was repeated three times independently with similar results. $P = 2.20\times10^{-3}$ (CD163) and $1.60\times10^{-3}$ (CD20).

**(c)** Representative immunohistochemical staining of CD163 (left) and CD20 (right) on paraffin-embedded tissue from the $T_{CyEM}$ and $T_{CM}$ groups. Scale bar = 50 µm. Results are representative of three different samples.

**(d)** Correlation analysis of the mRNA expression of *MS4A1* and *CD40LG* in an independent cohort of 49 tumor-stage MF patients. *P*-values were calculated using the Spearman rank correlation test.

**(e and f)** Immunofluorescence staining demonstrate that $FOXP3^+$ Tregs (green) in the $T_{CM}$ group exhibit higher expression of LAG3 (red) **(e)** and TIGIT (red) **(f)** compared with the $T_{CyEM}$ group. DAPI (blue) was used to visualize cell nuclei. Scale bar = 50 µm. Results are representative of three different samples.

**(g)** Multicolor IHC staining of tumor tissue samples show different TMEs in the two subgroups. (Panel 1) Granzyme $A^+$ $CD4^+$ tumorous cells were surrounded by abundant $CD163^+$ macrophages and $CD8^+$ T cells within the $T_{CyEM}$ group (exemplified by patient MF17), whereas only $CD8^+$ T cells were found in the surrounding microenvironment in the $T_{CyEM}$ group (exemplified by patient MF4). (Panel 2) Few $CD20^+$ B cells and $LAG3^+$ $FOXP3^+$ Tregs were found within the TME of the $T_{CyEM}$ group (exemplified by patient MF17), whereas $TOX^+$ tumorous cells were surrounded by abundant $CD20^+$ B cells and $LAG3^+$ $FOXP3^+$ Tregs in the $T_{CM}$ group (exemplified by patient MF28). DAPI (blue) was used to visualize cell nuclei. Scale bar = 100 µm. Results are representative of three different samples.

**Supplementary Table 1. M1 and M2 gene list.**

| **M1** | **M2** |
|---|---|
| CD86 | CSF1R |
| IL1B | IL1R2 |
| IRF1 | CTSA |
| CD40 | CTSB |
| IDO1 | CTSC |
| KYNU | TGFB1 |
| | CLEC7A |

**Supplementary Table 2. Sequencing analysis summary of 13 samples from 11 patients subject to WES.**

| Patient ID | Sample | Properly Mapped and Paired Reads (%) | Number of reads (millions) | Mean_ Depth | Tumor purity |
|---|---|---|---|---|---|
| MF4 | Tumor | 99.36% | 87.45 | 101.44 | 0.64 |
| | Blood | 99.25% | 78.88 | 99.09 | |
| MF6 | Tumor | 99.08% | 92.51 | 110.73 | 0.32 |
| | Blood | 99.19% | 85.5 | 100.63 | |
| MF14 | Tumor | 98.83% | 141.64 | 201.61 | 0.73 |
| | Blood | 98.66% | 78.56 | 108.46 | |
| MF15 | Tumor | 98.91% | 133.82 | 186.45 | 0.59 |
| | Blood | 98.63% | 65.17 | 89.88 | |
| MF17 | Tumor | 99.27% | 143.08 | 204.59 | 0.46 |
| | Blood | 99.19% | 94.92 | 132.21 | |
| MF21-1 | Tumor | 99.04% | 131.64 | 180.5 | 0.36 |
| | Blood | 99.21% | 81.41 | 111.17 | |
| MF21-2 | Tumor | 99.19% | 170.36 | 249.51 | 0.31 |
| | Blood | 99.21% | 81.41 | 111.17 | |
| MF22 | Tumor | 98.71% | 155.06 | 211.17 | 0.44 |
| | Blood | 98.51% | 77.17 | 102.99 | |
| MF26 | Tumor | 98.96% | 139.24 | 184.53 | 0.49 |
| | Blood | 99.09% | 76.68 | 108.09 | |
| MF28-1 | Tumor | 99.18% | 152.46 | 229.51 | 0.41 |
| | Blood | 98.97% | 76.52 | 112.34 | |
| MF30-1 | Tumor | 98.80% | 132.45 | 181.23 | 0.54 |
| | Blood | 98.63% | 73.14 | 103.37 | |
| MF30-2 | Tumor | 98.81% | 133.05 | 172.6 | 0.41 |
| | Blood | 98.63% | 73.14 | 103.37 | |
| P2 | Tumor | 98.75% | 194.42 | 256.37 | 0.21 |
| | Blood | 98.33% | 68.58 | 96.78 | |
