# Supplementary material for: Single-cell transcriptomics links malignant T cells to the tumor immune landscape in cutaneous T cell lymphoma
Source: Nat Commun. 2022 Mar 3;13:1158. doi: 10.1038/s41467-022-28799-3 (PMC8894386; doi:10.1038/s41467-022-28799-3)
Supplement: Supplementary file 5 — Reporting Summary [file 41467_2022_28799_MOESM5_ESM.pdf]

nature portfolio

Corresponding author(s): Fan Bai; Yang Wang

Last updated by author(s): Jan 28, 2022

Reporting Summary

Nature Portfolio wishes to improve the reproducibility of the work that we publish. This form provides structure for consistency and transparency in reporting. For further information on Nature Portfolio policies, see our Editorial Policies and the Editorial Policy Checklist.

Statistics

For all statistical analyses, confirm that the following items are present in the figure legend, table legend, main text, or Methods section.

n/a	Confirmed	
☐	☒	The exact sample size (*n*) for each experimental group/condition, given as a discrete number and unit of measurement
☐	☒	A statement on whether measurements were taken from distinct samples or whether the same sample was measured repeatedly
☐	☒	The statistical test(s) used AND whether they are one- or two-sided<br>*Only common tests should be described solely by name; describe more complex techniques in the Methods section.*
☒	☐	A description of all covariates tested
☐	☒	A description of any assumptions or corrections, such as tests of normality and adjustment for multiple comparisons
☐	☒	A full description of the statistical parameters including central tendency (e.g. means) or other basic estimates (e.g. regression coefficient) AND variation (e.g. standard deviation) or associated estimates of uncertainty (e.g. confidence intervals)
☐	☒	For null hypothesis testing, the test statistic (e.g. *F*, *t*, *r*) with confidence intervals, effect sizes, degrees of freedom and *P* value noted<br>*Give P values as exact values whenever suitable.*
☒	☐	For Bayesian analysis, information on the choice of priors and Markov chain Monte Carlo settings
☒	☐	For hierarchical and complex designs, identification of the appropriate level for tests and full reporting of outcomes
☒	☐	Estimates of effect sizes (e.g. Cohen's *d*, Pearson's *r*), indicating how they were calculated

Our web collection on statistics for biologists contains articles on many of the points above.

Software and code

Policy information about availability of computer code

Data collection	BD FACS ARIA II cell sorter (BD Biosciences)
Data analysis	After flow cytometry, single cell isolation was performed using FlowJo (v10.7.1). Analysis of single cell RNA-seq data was done using R (v4.0.3) with publicly available packages. Dimensionality reduction and differential gene expression was performed using the Seurat (v3.0.2) package. Plots were generated using the ggplot2 (v3.3.2) and GraphPad Prism (v 6.1) packages. Trajectory analysis was conducted using the Monocle 2 package (v2.16.0). Cell-cell interaction prediction was done using CellPhoneDB (v2.0). Non-negative matrix factorization analysis was performed by the R NMF package (v0.23.0). Phylogenetic trees were constructed using MEGA-X (v10.2.2).

For manuscripts utilizing custom algorithms or software that are central to the research but not yet described in published literature, software must be made available to editors and reviewers. We strongly encourage code deposition in a community repository (e.g. GitHub). See the Nature Portfolio guidelines for submitting code & software for further information.

Data

Policy information about availability of data

All manuscripts must include a data availability statement. This statement should provide the following information, where applicable:

- Accession codes, unique identifiers, or web links for publicly available datasets
- A description of any restrictions on data availability
- For clinical datasets or third party data, please ensure that the statement adheres to our policy

All relevant data are available in publicly accessible repositories. scRNA-seq reads were aligned to the hg19 reference genome, and scRNA-TCR data were aligned to the vdj-GRCh38 reference genome. The raw data from the scRNA-seq, TCR-seq and bulk exome sequencing experiments have been deposited in the Genome Sequence Archive for human (GSA-Human) under accession number HRA000166 [https://ngdc.cncb.ac.cn/gsa-human/browse/HRA000166]. The raw bulk RNA-seq

data have been deposited in the Gene Expression Omnibus database under accession numbers GSE168508 [https://www.ncbi.nlm.nih.gov/geo/query/acc.cgi?acc=GSE168508] and GSE109620 [https://www.ncbi.nlm.nih.gov/geo/query/acc.cgi?acc=GSE109620]. To gain access to the raw sequencing data from scRNA-seq, TCR-seq and bulk exome sequencing data, please submit requests to the GSA-Human online page for this study [https://ngdc.cncb.ac.cn]. The remaining data are available within the Article, Supplementary Information or Source Data file. The source data underlying Figs. 1c, g, 2e, g, 3j, k, 4d, 5c, 6a, c and Supplementary Fig. 3d are provided in the Source Data file.
Since October 2019, to comply with the "Guidance of the Ministry of Science and Technology (MOST) for the Review and Approval of Human Genetic Resources", we are required to deposit the genomic data of Chinese patients under a controlled access at the Genome Sequence Archive (GSA) in Beijing Institute of Genomics Data Center. As you can see here http://gsa.big.ac.cn/, The Genome Sequence Archive (GSA) is a data repository for archiving raw sequence reads. It accepts data submissions from all over the world and provides free access to global scientific communities. GSA is at the National Genomics Data Center, Beijing Institute of Genomics, Chinese Academy of Sciences. It is a famous, stable, and community supported data repository. Also, GSA is one of the recommended data repositories of Springer Nature publishing group.
In this work, most of the single cell sequencing data were collected after October 2019, so they have been deposited to GSA under a controlled access; some bulk sequencing data were collected before October 2019, so they could be deposited to GEO.

Field-specific reporting

Please select the one below that is the best fit for your research. If you are not sure, read the appropriate sections before making your selection.

☒ Life sciences ☐ Behavioural & social sciences ☐ Ecological, evolutionary & environmental sciences

For a reference copy of the document with all sections, see nature.com/documents/nr-reporting-summary-flat.pdf

Life sciences study design

All studies must disclose on these points even when the disclosure is negative.

Sample size	No standard methods were used to predetermine sample size. We used all the samples available in each dataset. A total of fifteen CTCL patients with informed consent were recruited from the Skin Lymphoma Clinic of Peking University First Hospital in the period from 2018 to 2019. Only patients with clear diagnoses, detailed clinical and pathological information were recruited in our study. The sample sizes are sufficient to provide stable single cell clustering results and to perform statistical analysis.
Data exclusions	No exclusion was applied to the uploaded raw data. For the final count matrix, we excluded cells based on pre-established criteria for single-cells: we excluded low quality samples and contaminating cells. Only genes detected in at least 10 cells were retained. We filtered out cells with fewer than 500 or more than 5,000 detected genes and those with a high mitochondrial content (>10%). The criteria are comprehensively detailed in the relevant Methods section.
Replication	The immunohistochemistry and immunofluorescence staining were performed on all samples and repeated at least three times. The staining results were calculated and verified by at least two researchers, and reproducible results were obtained.
Randomization	All patients who meet the criteria in our study were recruited in the cohort. No randomization was performed due to the nature of the study. In order to avoid batch effects, skin lesions were always processed in the same batch.
Blinding	The investigators handling the samples were not blinded during data collection and processing as the nature of cutaneous lymphoma was visible. Besides, blinding wasn't relevant as all individuals were compared to each other.

Reporting for specific materials, systems and methods

We require information from authors about some types of materials, experimental systems and methods used in many studies. Here, indicate whether each material, system or method listed is relevant to your study. If you are not sure if a list item applies to your research, read the appropriate section before selecting a response.

Materials & experimental systems

n/a	Involved in the study	
☐	☒	Antibodies
☐	☒	Eukaryotic cell lines
☒	☐	Palaeontology and archaeology
☒	☐	Animals and other organisms
☐	☒	Human research participants
☒	☐	Clinical data
☒	☐	Dual use research of concern

Methods

n/a	Involved in the study	
☒	☐	ChIP-seq
☐	☒	Flow cytometry
☒	☐	MRI-based neuroimaging

Antibodies

Antibodies used	CD4 (1:100, Abcam, ab133616, clone EPR6855), TCRα (1:100, H-1, santa, sc-515719), CD45RO (1:400, CST, 55618s, clone UCHL1), CD27 (1:500, Abcam, ab131254, clone EPR8569), granzyme A (1:100, Abcam, ab209205, clone EPR20161), granzyme B (1:800, CST, 17215s, clone D2H2F), perforin (1:10, eBioscience, 14-9994-82, clone deltaG9), granulysin (1:250, Abcam, ab241333, clone

EPR22110-101), TOX (1:300, Abcam, ab237009, clone NAN448B), LGALS9 (1:100, Abcam, ab227046, clone EPR22214), CD40 (1:250, Abcam, ab224639, clone EPR20735), CD40L (1:100, Abcam, ab257319, clone CD40LG/2761), CD20 (1:50, Abcam, ab78237, clone EP459Y), FOXP3 (1:100, Abcam, ab20034, clone 236A/E7), LAG3 (1:1000, Abcam, ab209236, clone EPR20261), CD163 (1:500, Abcam, ab182422, clone EPR19518), CD8 (1:1000, Proteintech, 66868-1-1g, clone 1G2B10) and CD45RA (1:300, Millipore, 05-1413, clone MEM 56), TIGIT (1:500, CST, 99567T, clone E5Y1W),CD45-PE(1:1000, Invitrogen, 12-0459-42, clone HI30) .

Validation

All the antibodies used in this study were commercial antibodies and were only used for applications, with validation procedures described on the following sites of the manufactures:
https://www.abcam.cn/cd4-antibody-epr6855-ab133616.html
https://www.scbt.com/p/tcr-alpha-antibody-h-1?requestFrom=search
https://www.cellsignal.com/products/primary-antibodies/cd45ro-uchl1-mouse-mab-ihc-specific/55618
https://www.abcam.cn/cd27-antibody-epr8569-ab131254.html
https://www.abcam.cn/granzyme-a-antibody-epr20161-ab209205.html
https://www.cellsignal.com/products/primary-antibodies/granzyme-b-d2h2f-rabbit-mab/17215
https://www.thermofisher.com/antibody/product/Perforin-Antibody-clone-dG9-delta-G9-Monoclonal/14-9994-82
https://www.abcam.cn/gnlygranulysin-antibody-epr22110-101-ab241333.html
https://www.abcam.cn/tox-antibody-nan448b-ab237009.html
https://www.abcam.cn/galectin-9gal-9-antibody-epr22214-ab227046.html
https://www.abcam.cn/cd40-antibody-epr20735-ab224639.html
https://www.abcam.cn/trapcd40l-antibody-cd40lg2761-ab257319.html
https://www.abcam.cn/cd20-antibody-ep459y-ab78237.html
https://www.abcam.cn/foxp3-antibody-236ae7-ab20034.html
https://www.abcam.cn/lag-3-antibody-epr20261-ab209236.html
https://www.abcam.cn/cd163-antibody-epr19518-ab182422.html
https://www.ptglab.com/products/CD8A-Antibody-66868-1-Ig.htm
https://www.sigmaaldrich.cn/CN/zh/product/mm/051413
https://www.cellsignal.com/products/primary-antibodies/tigit-e5y1w-xp-rabbit-mab/99567
https://www.thermofisher.com/antibody/product/CD45-Antibody-clone-HI30-Monoclonal/12-0459-42?imageId=89487

Eukaryotic cell lines

Policy information about cell lines

Cell line source(s)

MJ, PB2B, Myla, Hut78 and SZ4 were generous gifts from Youwen Zhou (Department of Dermatology and Skin Science, University of British Columbia); Mac-1A and Mac-2A were generous gifts from Marshall E. Kadin (School of Medicine, Boston University).

Authentication

Cell lines were authenticated by short tandem repeat (STR) profiling.

Mycoplasma contamination

We confirmed that all cell lines were negative for mycoplasma contamination.

Commonly misidentified lines
(See ICLAC register)

No commonly misidentified cell lines were used.

Human research participants

Policy information about studies involving human research participants

Population characteristics

Sample ID Gender Status Age Diagnosis TNMB Stage
MF4 male Dead 71 CD4+ Mycosis fungoides(MF) T3N3M0B0
MF6 female Dead 79 CD8+ MF T3N0M0B0
MF7 female Alive 56 CD4+ MF T3N1M0B0
MF14 female Alive 31 CD8+ MF T2bN1M0B0
MF15 male Alive 35 CD4+ MF T2bN0M0B0
MF17 male Alive 36 CD4+ MF T3N1M0B0
MF18 male Alive 70 CD4+ MF T3N3M0B1
MF21 male Alive 79 CD4+ MF T3N2M0B0
MF22 male Alive 71 CD4+ MF T2bN0M0B0
MF26 male Alive 15 CD4+ MF T2bN1M0B0
MF27-1 female Alive 57 CD4+ MF T3N1M0B0
MF28 male Alive 60 CD4+ MF T4N2M0B1
MF30 female Alive 48 CD4+ MF T3N1M0B0
pcALCL female Alive 67 CD8+ primary cutaneous anaplastic large cell lymphoma(pcALCL) T2bN0M0B0
pcALCL2 female Alive 41 CD4+ pcALCL T2bN0M0B0

Recruitment

All recruited patients who fulfilled the 2018 WHO/EORTC classification criteria were recruited in our study. All diagnoses were verified by at least two dermatopathologists according to the criteria. Only patients with clear diagnoses, detailed clinical and pathological information were included. Patients were selected if they were treatment naïve or did not receive anti-lymphoma therapy at least 6 months prior to the biopsy. Finally, a total of fifteen CTCL patients with informed consent were recruited from the Skin Lymphoma Clinic of Peking University First Hospital in the period from 2018 to 2019.

Ethics oversight

All participants provided written consent for specimen collection and analysis under the study protocol approved by the

Ethics oversight | Peking University First College Hospital Ethics Committee. We have complied with all relevant ethical regulations for work with human participants.

Note that full information on the approval of the study protocol must also be provided in the manuscript.

Flow Cytometry

Plots

Confirm that:

☒ The axis labels state the marker and fluorochrome used (e.g. CD4-FITC).

☒ The axis scales are clearly visible. Include numbers along axes only for bottom left plot of group (a 'group' is an analysis of identical markers).

☒ All plots are contour plots with outliers or pseudocolor plots.

☒ A numerical value for number of cells or percentage (with statistics) is provided.

Methodology

Sample preparation	Each fresh tissue sample used for tissue dissociation was placed in a 10-mL sterile Petri dish with cold phosphate-buffered saline (PBS) (Sigma-Aldrich, P5119) and washed three times. The tissue was minced into pieces (<1 mm3) on ice and placed into 10 mL serum-free RPMI-1640 media supplemented with 200 U/mL collagenase (Sigma-Aldrich, C9891), 10 mM HEPES (Gibco, 15630-080), 1mM Na-Pyruvate (Gibco, 11360-070), 0.01% DNase (BioLabs, M0303S), 1% penicillin/streptomycin (Sigma-Aldrich) and 25 μg/mL gentamicin (Sigma-Aldrich, G1914) within minimum ischemic time. To obtain single-cell suspensions with a high viability rate, the solution was transferred into a gentleMACS C Tube (Miltenyi Biotec, 130-093-237) and placed into a gentleMACS octo dissociator (Miltenyi Biotec, 130-095-937), in which it was incubated for 45 min at 37 ? according to the manufacturer's instructions. After digestion, the suspension was filtered using a Falcon 40-μm cell strainer (Corning, 352340) and gently added to a new 15-mL centrifuge tube with 10 mL PBS. Next, 10 μL cell suspension was used to confirm the cell viability with trypan blue staining (Solarbio, C0040). The sample was then centrifuged at 110 × g for 10 min and the supernatant was discarded. The cell pellet was resuspended with 200 μL cold serum-free PBS to prepare it for cell staining and flow cytometry.
Instrument	The single-cell suspension was sorted using a MoFlo Astrios (Beckman Coulter).
Software	FlowJo software (v10.7.1, BD Inc, USA) was used to analyze flow cytometry data.
Cell population abundance	We ensured that the initial gate was used to sort a cell count of 100,000 cells and collected the relevant cell population.
Gating strategy	Samples were compensated by single color stains. To remove doublets, single cells were gated by standard forward scatter height (FSC-H) versus width (FSC-W) and side scatter height (SSC-H) versus width (SSC-W). We sorted the 7-AAD- population to obtain viable cells. We sorted all T cell populations within the CD45+CD3+ gate. To elucidate the tumor microenvironment, we also sorted other subtypes disproportionally, including the non-T immune cell population (CD45+CD3-) and non-immune cell population (CD45-).

☒ Tick this box to confirm that a figure exemplifying the gating strategy is provided in the Supplementary Information.
